# DNA methylation and chromatin accessibility profiling of mouse and human fetal germ cells

**DOI:** 10.1038/cr.2016.128

**Published:** 2016-11-08

**Authors:** Hongshan Guo, Boqiang Hu, Liying Yan, Jun Yong, Yan Wu, Yun Gao, Fan Guo, Yu Hou, Xiaoying Fan, Ji Dong, Xiaoye Wang, Xiaohui Zhu, Jie Yan, Yuan Wei, Hongyan Jin, Wenxin Zhang, Lu Wen, Fuchou Tang, Jie Qiao

**Affiliations:** 1 Department of Obstetrics and Gynecology Third Hospital, Biomedical Institute for Pioneering Investigation via Convergence & Center for Reproductive Medicine, College of Life Sciences, Peking University, Beijing 100871, China; 2 Key Laboratory of Assisted Reproduction, Ministry of Education, Beijing 100191, China; 3 Beijing Key Laboratory of Reproductive Endocrinology and Assisted Reproductive Technology, Beijing 100191, China; 4 National Institute of Biological Sciences, Beijing 102206, China; 5 Ministry of Education Key Laboratory of Cell Proliferation and Differentiation, Beijing 100871, China; 6 Beijing Advanced Innovation Center for Genomics, Beijing 100871, China; 7 Peking-Tsinghua Center for Life Sciences, Peking University, Beijing 100871, China

**Keywords:** chromatin, epigenetics, transcription, stem cell biology, development

## Abstract

Chromatin remodeling is important for the epigenetic reprogramming of human primordial germ cells. However, the comprehensive chromatin state has not yet been analyzed for human fetal germ cells (FGCs). Here we use nucleosome occupancy and methylation sequencing method to analyze both the genome-wide chromatin accessibility and DNA methylome at a series of crucial time points during fetal germ cell development in both human and mouse. We find 116 887 and 137 557 nucleosome-depleted regions (NDRs) in human and mouse FGCs, covering a large set of germline-specific and highly dynamic regulatory genomic elements, such as enhancers. Moreover, we find that the distal NDRs are enriched specifically for binding motifs of the pluripotency and germ cell master regulators such as NANOG, SOX17, AP2γ and OCT4 in human FGCs, indicating the existence of a delicate regulatory balance between pluripotency-related genes and germ cell-specific genes in human FGCs, and the functional significance of these genes for germ cell development *in vivo*. Our work offers a comprehensive and high-resolution roadmap for dissecting chromatin state transition dynamics during the epigenomic reprogramming of human and mouse FGCs.

## Introduction

Two waves of genome-wide reprogramming of DNA methylation occur during mammalian embryonic development, and have been shown to be crucial for the proper development of mammals^1,2,3,4^. Very recently, the dynamic transcriptome and DNA methylome of human fetal germ cells (FGCs) during development have been comprehensively analyzed by our and other groups^5,6,7^. Approximately 10-11 weeks after gestation, the global DNA methylation levels of human FGCs reach the lowest point; the entire genome is nearly devoid of DNA methylation, with only 6%-7% (median level) residual methylation left in the genome. On the other hand, the major families of repetitive elements such as long-interspersed nuclear elements (LINEs), short-interspersed nuclear elements (SINEs) and α satellites still retain abundant residual DNA methylation (∼12%-37%), which may provide a basis for potential transgenerational epigenetic inheritance. Despite the global drastic DNA methylation erasure, FGCs maintain relatively stable transcriptome between 4 and 11 weeks after gestation.

Although the global and thorough DNA demethylation patterns of human FGCs have been revealed, the accompanying chromatin states in human germline remains unexplored. Mouse is a well-established model for the study of mammalian embryology, and parallel comparison between mouse and human samples can be very informative and lead to a better understanding of human embryogenesis. Several groups have applied the chromatin immunoprecipitation coupled with high-throughput sequencing (ChIP-seq) strategy to mouse primordial germ cells (PGCs) at several developmental time points during sexual differentiation and meiotic initiation, and provided the genome-wide histone modification profiles of mouse PGCs^8,9,10^.

Furthermore, recent studies have reported that mouse embryonic stem cells/induced pluripotent stem cells can be induced into epiblast-like cells, which can be further programmed into PGC-like cells (PGCLCs) with capacity for gametogenesis, thus offering a robust system for the investigation of key features of mouse germ cell specification and development *in vitro*^11,12,13,14,15^. Moreover, the researchers have applied ChIP-seq to this *in vitro* system and analyzed reprogramming of histone modification during PGC specification and development, which is in agreement with the previous immunostaining results^16,17,18^.

Although the genome-wide histone modification landscapes of mouse *in vivo* germ cells and *in vitro* PGCLCs have been profiled and several germline-specific properties of epigenetic reprogramming have been revealed, the study of genome-scale chromatin states in human FGCs is still challenging, due to the scarcity of materials and technical difficulties. Recently, nucleosome occupancy and methylation sequencing (NOMe-seq) technique has been developed, which utilizes the M.CviPI GpC methyltransferase to specifically methylate the GpC dinucleotides in open chromatin regions^19,20^. On the basis of this principle, NOMe-seq can dissect the chromatin accessibility, as well as endogenous DNA methylation from target cell types, even from a limited number of cells. Here we used NOMe-seq technique to analyze human FGCs as well as their neighboring somatic cells in the gonads of postimplantation embryos. In parallel, we also analyzed mouse FGCs and somatic cells at comparable developmental time points to dissect the evolutionarily conserved as well as species-specific features of DNA methylome and chromatin states of the genome of human germline.

## Results

### NOMe-seq of the human and mouse gonadal germ cells

We sorted KIT-positive gonadal FGCs from six embryos between 7 and 26 weeks of human gestation using magnetic-activated cell sorting (MACS) or fluorescence-activated cell sorting (FACS) (Materials and Methods). In parallel, we also isolated GFP-positive PGCs from the GOF (OCT4-GFP transgenic mice with proximal enhancer deleted) embryos at embryonic day (E) 11.5, E13.5 and E16.5, which are the key time points for epigenome reprogramming of mouse PGCs. To better understand the relationship between FGCs and their niche cells, we also collected KIT-negative and GFP-negative gonadal somatic cells (Soma) from these human and mouse embryos, respectively. We performed NOMe-seq and RNA-seq on all these samples, and in total generated 1.63 Tb of sequencing data for the subsequent analysis. On average for each NOMe-seq sample, we sequenced 37.8 Gb data (Materials and Methods and Supplementary information, Table S1). For NOMe-seq, we have at least two independent biological or technical replicates for most developmental stages, which show highly reproducible patterns (Supplementary information, Figures S1, S2 and Table S1). The efficiency of M.CviPI GpC methyltransferase was reasonably high (93.1% in human cells; 93.2% in mouse cells), and the bisulfite conversion rate was 98.7% on average (98.8% and 98.5% in human and mouse cells, respectively), which collectively demonstrate the accuracy and high sensitivity of this method when applied to mouse and human germ cells (Supplementary information, Figure S3 and Table S1).

From the sequencing data of NOMe-seq, we obtained genome-wide maps of nucleosome occupancy and endogenous DNA methylation in the corresponding regions. GCH sites (GCA/GCT/GCC) were used to analyze chromatin accessibility, while the WCG sites (ACG/TCG) were used to analyze the endogenous DNA methylation^19^. From these analyses, we integrated the epigenome landscapes of the chromatin accessibility and DNA methylome to gain a comprehensive understanding of the epigenetic reprogramming of the mammalian germline *in vivo*.

### Endogenous DNA methylation reprogramming in mammalian germline

**Figure 1:**
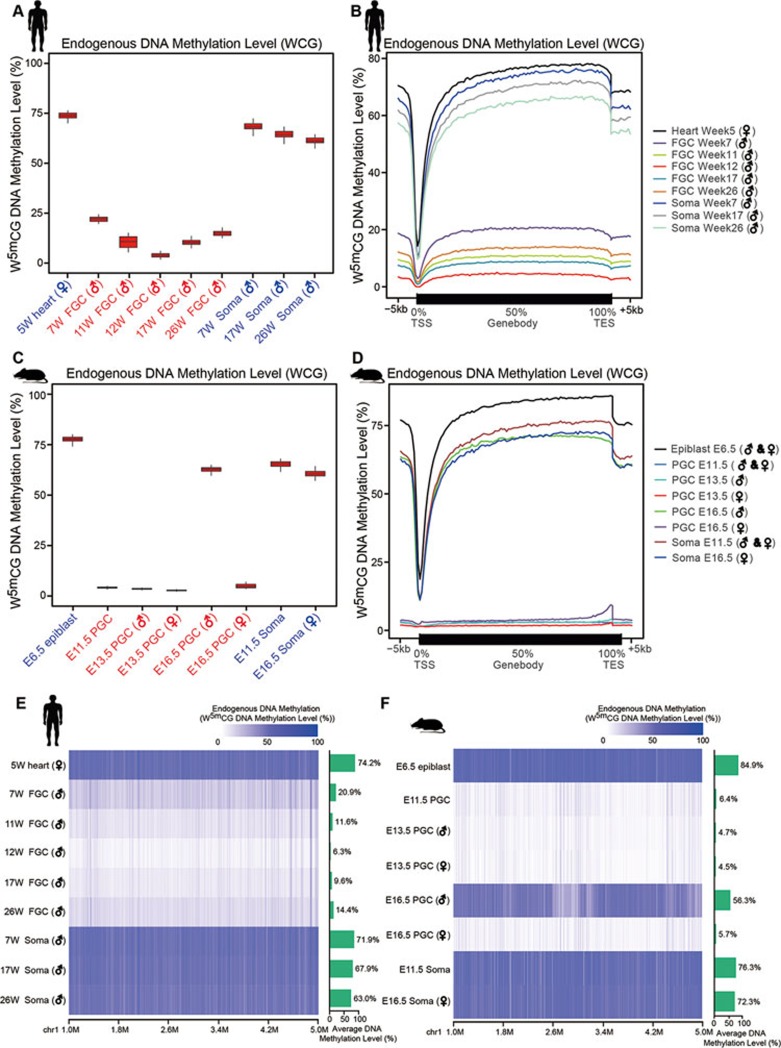
Endogenous DNA methylation reprogramming in mammalian germline. **(A)** Boxplots showing the average endogenous DNA methylation dynamics in human germline. Endogenous DNA methylation level was calculated using the WCG sites (ACG and TCG trinucleotides). **(B)** Endogenous DNA methylation distribution along the gene body regions and their flanking regions in human fetal germ cells and somatic cells, with the DNA methylation level decreasing at TSS and increasing along the gene body region, and then decreasing at transcription end site (TES). **(C)** Boxplots showing the average endogenous DNA methylation dynamics in mouse germline. **(D)** Endogenous DNA methylation distribution along the gene body regions and their flanking regions in mouse PGCs and somatic cells. **(E**, **F)** The heatmap views of a representative section of chromosome 1 showing the dynamics of DNA methylome in human **(E)** and mouse **(F)** germline. Color key from white to dark blue indicates the endogenous DNA methylation level from low to high.

We first segregated the ACG and TCG trinucleotides of the human genome, and calculated methylation level of the cytosine in the center position to estimate the endogenous DNA methylation levels of each sample. We found that in the early postimplantation embryos (heart tissue of 5-week embryo), the average DNA methylation level is as high as 72.9% (*n* = 2), whereas in the 7-week male FGCs, the methylation level decreases drastically to 22.2% (*n* = 2); in 12-week male FGCs, the DNA methylation level reaches its lowest point at only 6.7% on average (*n* = 2), confirming that germline DNA demethylation occurs within the first 12 weeks of human development, consistent with our previous results^5^. After 12 weeks, there is a small but significant increase in DNA methylation level in male FGCs, reaching to 10.7% (*n* = 2) at 17 weeks, and 15.1% (*n* = 2) at 26 weeks, indicating a global remethylation in the male germline takes place after 12 weeks of human gestation (Figure 1A and 1B).

We also analyzed the DNA methylation dynamics in mouse PGCs in parallel. The average DNA methylation level falls from 77.7% in E6.5 epiblast to 5.0% in E11.5 PGCs, and further decreases to 4.2% and 3.8% in E13.5 male and female PGCs, respectively. This extremely hypomethylated state is maintained in E16.5 female PGCs (5.6%), whereas in E16.5 male PGCs, the DNA methylation level recovers to 61.4% (Figure 1C and 1D).

Despite drastic changes in DNA methylation in the human and mouse germline, the average DNA methylation level of neighboring somatic cells maintains at a high level (64.7%), which is consistent with previous reports^5,6,7,21,22^ and verifies the accuracy of our analysis (Figure 1).

We further investigated DNA methylation dynamics and chromatin accessibility landscapes on chromosome X (chrX) to obtain a better understanding of epigenetic reprogramming of the sex chromosome, and we included one of the autosomes, chromosome 1 (chr1) as the control. Intriguingly, both chrX and chr1 clearly undergo global DNA methylation resetting during human and mouse germ cell development, with chrX having a slightly higher DNA methylation level than autosomes in all human male germ cells and most mouse PGCs, except in E16.5 male PGCs. ChrX is slightly less accessible than autosomes in both human and mouse germ cells. Higher DNA methylation level and relatively closed chromatin state suggest that epigenetic reprogramming is slightly less extensive on chrX than on autosomes during the period of germ cell development (Supplementary information, Figure S4).

### Chromatin accessibility of the promoter regions in mammalian germline

**Figure 2:**
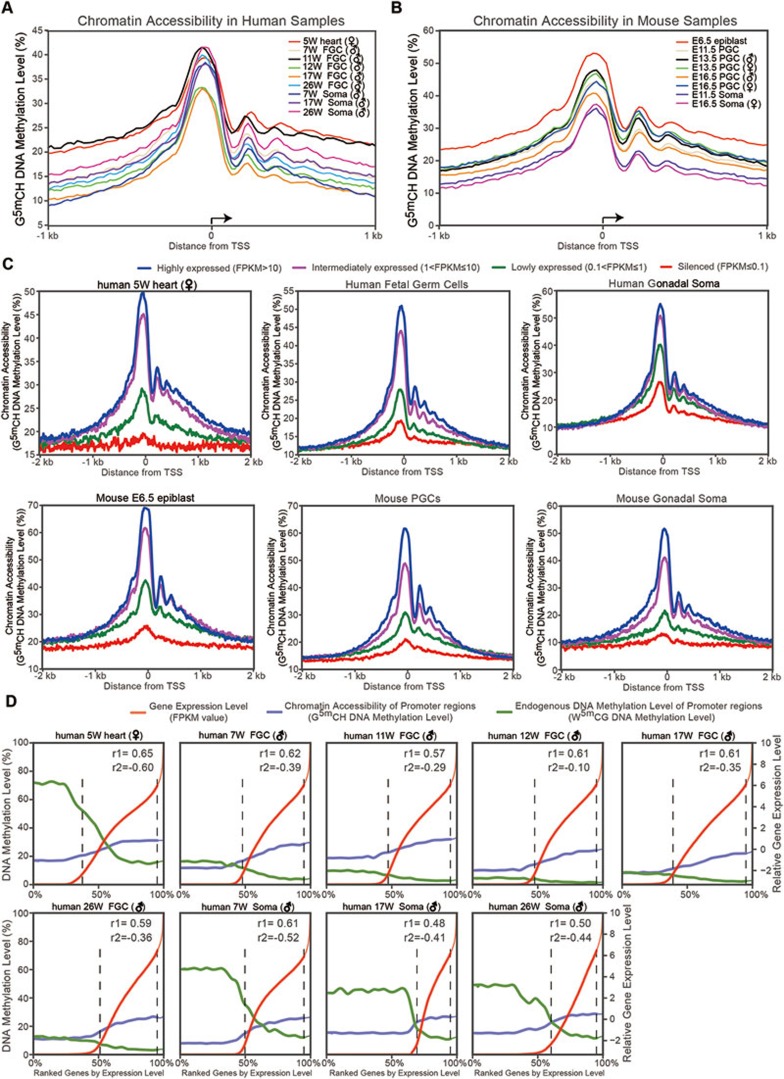
Chromatin accessibility of the promoter regions in mammalian fetal germ cells. **(A**, **B)** Chromatin accessibility patterns of the promoter regions in human **(A)** and mouse **(B)** samples. Chromatin accessibility is calculated using the DNA methylation level of the GCH sites (GCA, GCT and GCC trinucleotides). **(C)** Relationship between chromatin accessibility of the promoter regions and corresponding protein-coding RefSeq gene expression in human and mouse samples. Genes are classified into four groups according to their expression level. Human fetal germ cells and mouse PGCs are presented in the middle panel as the average signals of all the human germ cells from 7 to 26 weeks of gestation and all of the mouse PGCs from E11.5 to E16.5, respectively. **(D)** The Spearman correlation (*r1*) between the gene expression level (red) and chromatin accessibility of the promoter regions (light blue), and the Spearman correlation (*r*2) between the gene expression level (red) and endogenous DNA methylation level of the promoter regions (green) in human fetal germ cells and somatic cells; the horizontal axis from left to right represents the genes with their expression level from low to high.

To investigate comprehensively nucleosome occupancy and chromatin accessibility of the mammalian germline we applied a customized bisulfite sequencing analysis pipeline on our NOMe-seq data (Materials and Methods) and identified, in total, 116 887 and 137 557 nucleosome-depleted regions (NDRs) in human and mouse FGCs, respectively. 32 053 and 28 293 NDRs are located in the promoter regions of RefSeq genes (proximal NDRs) in human and mouse FGCs, respectively (Supplementary information, Table S2). As expected, the nucleosome-depleted signals are strongly enriched in the promoter regions, especially at the transcription start site (TSS), indicating high accessibility of the promoters of actively transcribing genes to facilitate the recruitment of transcriptional machinery and some regulatory transcription factors (TFs) to the TSS region (Figure 2A and 2B). More importantly, downstream of the TSS, we can clearly identify at least three nucleosome positioning signals with periodical spacing (+1, +2 and +3 nucleosome position), whereas upstream of the TSS, the nucleosomes are weakly phased. This asymmetrical distribution of the nucleosome spacing surrounding the TSSs indicates that nucleosome patterning may be involved in regulating transcriptional orientation of RNA polymerase stalling and elongation. It is also possible that these regularly positioned nucleosomes immediately downstream of the TSSs are important for repressing undesired transcription initiation from alternative TSSs of the same gene^23,24,25^.

To further investigate the relationship between promoter accessibility and transcriptional activity, we grouped genes into highly expressed (RPKM > 10), intermediately expressed (1 < RPKM ≤ 10), lowly expressed (0.1 < RPKM ≤ 1) and silenced genes (RPKM ≤ 0.1) according to their expression levels. We found that promoter accessibility is highly correlated with the expression level of corresponding genes, with the promoter regions of highly expressed genes exhibiting the highest chromatin accessibility, whereas those of lowly expressed or silenced genes having lower chromatin accessibility and relatively closed chromatin states (Figure 2C and Supplementary information, Figures S1, S2). The highly expressed genes also have strongly phased nucleosomes at +1 and +2 positions downstream of the TSS, whereas genes with low expression levels are likely to have weakly phased or even evicted nucleosomes at +1 position (Figure 2C). All cells, including FGCs and somatic cells in human and mouse share similar global organization of nucleosomes in the promoter regions described above, which demonstrates evolutionary conservation of the nucleosome patterning at TSSs and its potential impact to the gene expression divergence between germ cells and somatic cells, in agreement with the previous publications^25,26,27^. Besides the protein-coding RefSeq genes, these patterns are also observed in long-non-coding RNAs (lncRNAs). We quantified the expression levels of known lncRNAs in the human and mouse FGCs using Gencode datasets, and found highly expressed lncRNAs also have the more open promoter and more strongly phased nucleosomes downstream of the TSS regions, whereas lncRNAs with lower transcriptional activities tend to have closed promoters and weakly phased nucleosomes surrounding the TSS regions (Supplementary information, Figure S5A). These results collectively indicate that the openness of the promoter regions can indicate the transcriptional activity of genes (regardless of whether they have protein coding potential or not) in the mammalian germline during the period when the genome is nearly free of DNA methylation.

In parallel, we compared the endogenous CpG methylation levels in the promoter regions and the expression levels of the corresponding genes, and found, expectedly, a negative correlation in both FGCs and somatic cells at different developmental stages, including stage when the endogenous CpG methylation is reset to a very low level (Figure 2D and Supplementary information, Figure S5B and S5C).

### Chromatin accessibility of the distal regulatory elements in mammalian germline

**Figure 3:**
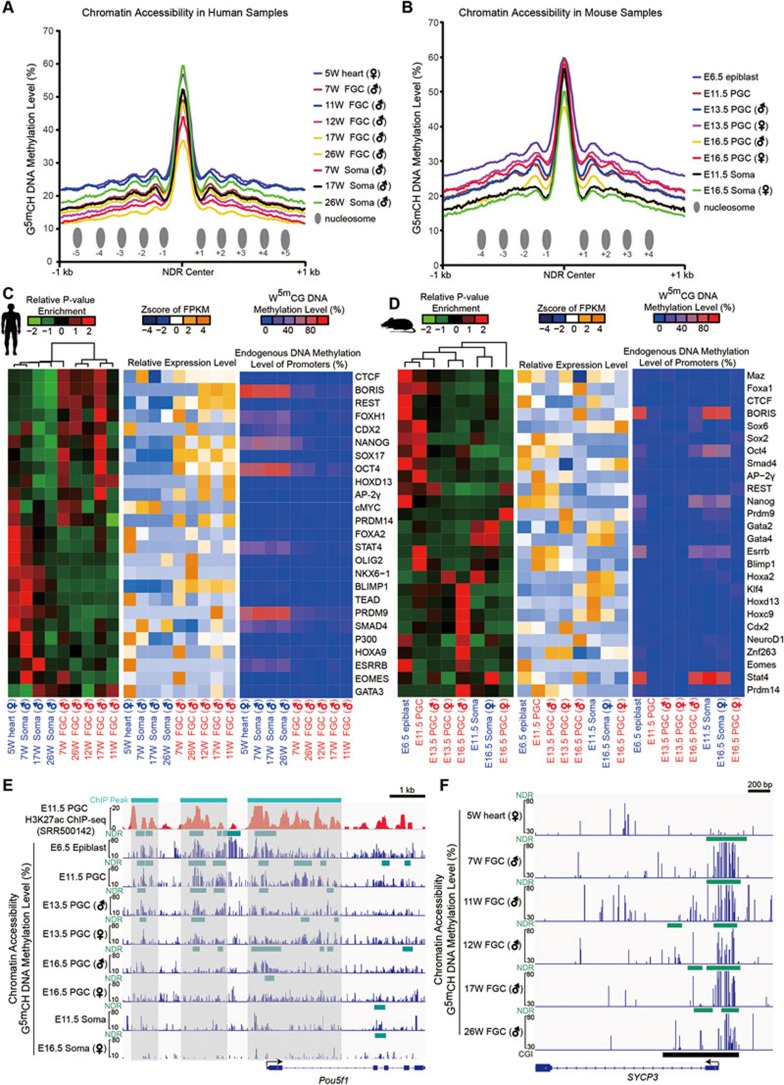
Chromatin accessibility of the distal regulatory elements in mammalian germline. **(A**, **B)** Chromatin accessibility patterns of NDRs and the flanking regions showing 4-5 symmetrically positioned nucleosomes in human **(A)** and mouse **(B)** samples. **(C**, **D)** Heatmaps showing the enrichment patterns, the corresponding relative gene expression levels and the endogenous DNA methylation levels of the promoter regions of the known transcription factors of the human **(C)** and mouse **(D)** distal NDRs. Color key from green to red indicates the enrichment from weak to strong; from dark blue to yellow indicates the gene expression level from low to high; from blue to red indicates the DNA methylation level from low to high. **(E**, **F)** Representative genome browser snapshots of the NOMe-seq signal in *Pou5f1* **(E)** and *SYCP3* **(F)** loci. E11.5 H3K27ac ChIP-seq data are from^10^. Blue bars in **(E)** and **(F)** indicate DNA methylation levels of GCH sites calculated based on NOMe-seq data sets.

More than half of the NDRs we identified are distal NDRs (located at least 2 kb away of the annotated TSS). Different from the proximal NDRs, accessibility of the distal NDRs is weakly correlated with the gene expression levels of their neighboring genes (data not shown). Distal NDRs are symmetrically surrounded by 3-5 regularly spaced nucleosomes with intervals ranging from 160 to 280 bp (Figure 3A and 3B). Most of the distal regulatory elements, such as enhancers, are likely to recruit tissue-specific TFs to regulate expression of their target genes^28,29,30^. We took full advantage of the distal NDRs we identified to analyze the preferential enrichment patterns of TF motifs in FGCs and their surrounding somatic cells in human and mouse. We found that distal NDRs in human FGCs are more likely to be enriched for binding motifs of TFs such as NANOG, SOX17, AP2γ (also called TFAP2C) and OCT4 (also called POU5F1) (Figure 3C). This indicates that these pluripotency-specific or early germ cell-specific TFs are functionally important for human FGC development potentially by setting an open chromatin state in a large set of downstream target genes. On the other hand, distal NDRs in human gonadal somatic cells have a strong enrichment for the binding motifs of FOXA2, STAT4, NKX6-1, OLIG2 and TEAD (Figure 3C). This suggests that these TFs play crucial roles in initiating and maintaining open chromatin states for somatic cell-specific genes and consequently impact on gonadal somatic cell development. Furthermore, Ap2γ, Sox2, Oct4, Smad4, Esrrb and Klf4 binding motifs are enriched in the distal NDRs of mouse PGCs and E6.5 epiblast, whereas Gata2, Gata4 and Hoxa2 binding motifs are enriched in the distal NDRs of mouse gonadal somatic cells (Figure 3D-3F). This is compatible with the fact that Sox2 is highly expressed in early mouse PGCs and is crucial for their development, whereas SOX17 is highly expressed in early human PGCs and is important for their development^7,31,32^. This also suggests that beyond the general evolutionarily conserved characteristics of the chromatin states, there are also distinct features between human and mouse germ cell development. More importantly, human FGCs from 7- to 26-week male embryos share similar TF-enrichment patterns of distal NDRs and are clustered together in an unsupervised hierarchical clustering analysis, whereas gonadal somatic cells and heart tissue at early postimplantation stage are clustered together (Figure 3C). Similarly, most of the mouse PGCs are clustered together with E6.5 epiblast, except the E16.5 female PGCs, which possess a relatively unique TF-enrichment pattern, suggesting that after entering meiotic arrest, mouse E16.5 female PGCs acquire very striking meiosis-related features in chromatin states (Figure 3D).

### Chromatin accessibility of imprinted genes and germline-specific genes

**Figure 4:**
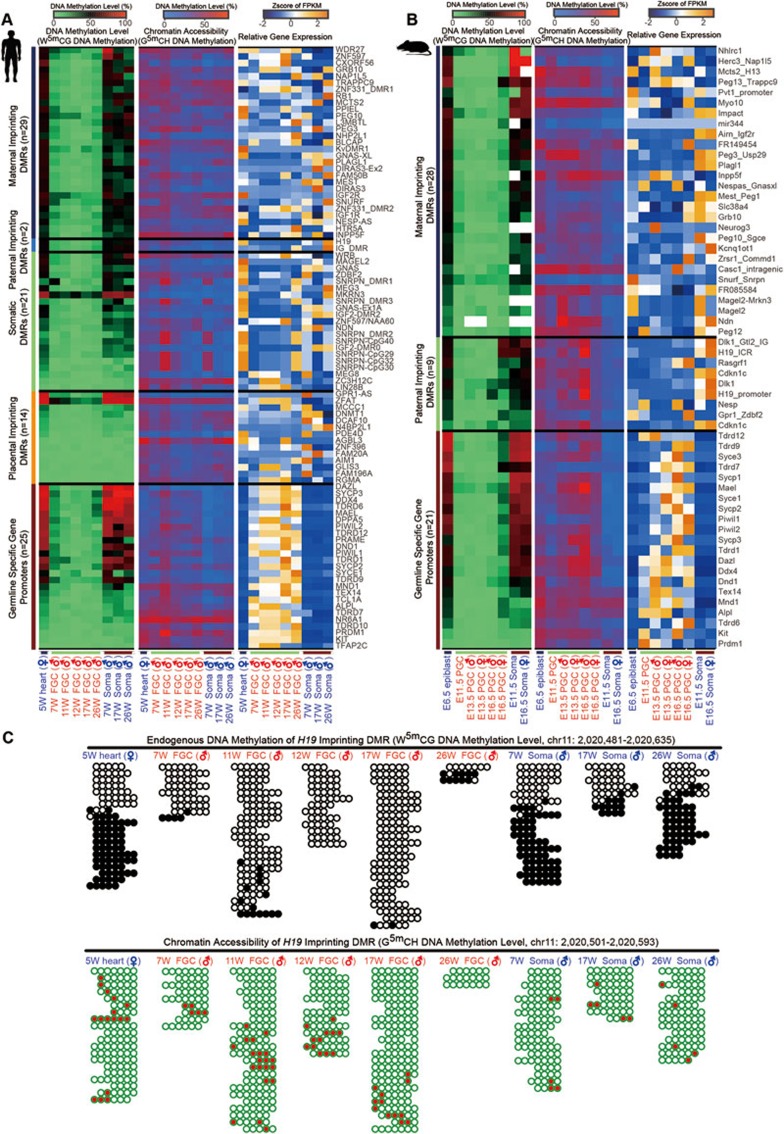
Chromatin accessibility landscapes of imprinted genes and germline-specific genes. **(A**, **B)** Heatmaps showing endogenous DNA methylation (WCG DNA methylation level), chromatin accessibility of the promoter regions (GCH DNA methylation level) and relative gene expression (*z*-score of the FPKM values) of human **(A)** and mouse **(B)** imprinted genes and germline-specific genes. Notably, in the heatmaps, the endogenous DNA methylation levels of human and mouse imprinted genes were calculated based on the WCG DNA methylation levels of the imprinting DMRs, whereas the endogenous DNA methylation levels for the germline-specific genes were calculated based on the WCG DNA methylation levels of their promoter regions. **(C)** DNA methylation graphs showing endogenous DNA methylation dynamics (upper panel) and chromatin accessibility (bottom panel) of human *H19* imprinted DMR across stages. In upper panel, white and open cycles indicate the unmethylated CpG sites in WCG context (endogenously unmethylated state), whereas the black and filled cycles indicate the methylated CpG sites in WCG context (endogenously methylated state). In the bottom panel, green and open cycles (the unmethylated GCH sites) indicate closed chromatin, whereas the red and filled cycles (the methylated GCH sites) indicate opened chromatin. Only the pair-ended reads with no less than four consecutive WCG or GCH trinucleotides covered are plotted.

We and others previously reported that DNA methylation in the known imprinted differentially methylated regions (DMRs) is erased during the global DNA demethylation event in human and mouse FGCs^5,6,7^, but little is known about chromatin accessibility of these DMRs. Our NOMe-seq results now show that unlike DNA methylation, chromatin accessibility of the imprinted DMRs is comparable before and after DNA demethylation (Figure 4A-4C).

Moreover, we found that DNA methylation levels of the promoter regions of germline-specific genes, which are highly and specifically expressed in FGCs, are significantly lower in FGCs than in the neighboring somatic cells in the gonad, whereas chromatin accessibility of most of these promoter regions is significantly higher in FGCs than in the somatic cells. Interestingly, the promoter of some germ cell-specific genes is also in an open state in 5-week heart tissue, which indicates that chromatin of some lineage-restricted genes is already made accessible at an earlier developmental stage preparing for the flexible regulation of their expression (Figures 3F and 4A-4C).

### Distinct features of chromatin accessibility in mammalian germline

**Figure 5:**
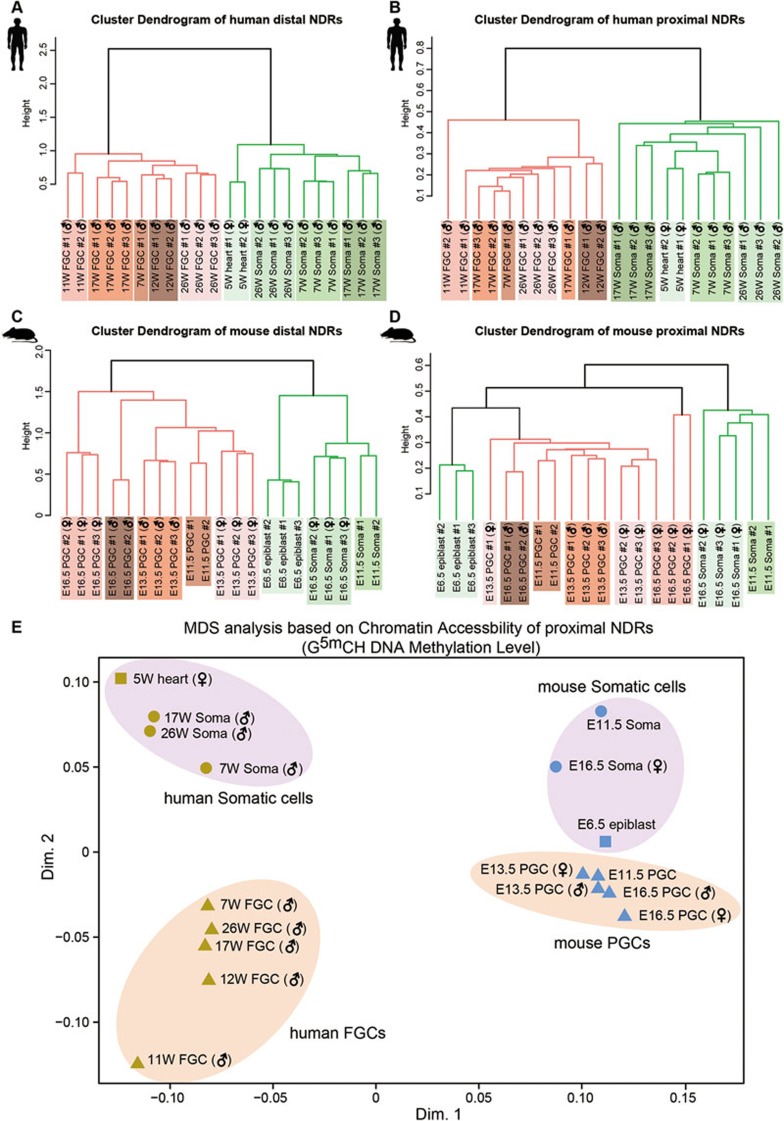
Distinct features of chromatin accessibility in mammalian germline. **(A**-**D)** Unsupervised hierarchical clustering analysis of chromatin accessibility of distal and proximal NDRs in human and mouse samples across replicates. **(E)** Multidimensional scaling (MDS) analysis of chromatin accessibility of the proximal NDRs of human-to-mouse homologous regions.

We next explored unique features of chromatin accessibility existing in the mammalian FGCs by systematically comparing the accessibility of distal and proximal NDRs in human and mouse FGCs and somatic cells. From the hierarchical clustering analysis and principle component analysis (PCA), we find that both the distal and proximal NDRs can accurately separate FGCs and somatic cells, with FGCs at different time points clustered together whereas somatic cells grouped together and separately from FGCs, implying that chromatin accessibility patterns of FGCs are distinct from somatic cells. Furthermore, during the DNA demethylation process, chromatin status of FGCs is kept relatively stable, compatible with the fact that during early FGC development from 4 to 11 weeks, the transcriptome of the germ cells is relatively stable despite the global demethylation of the genome^5,6,7^ (Figure 5A-5D and Supplementary information, Figure S6A-S6D).

We also calculated signal intensity of the distal and proximal NDRs and only took the human-mouse homologous regions for comparison. Multidimensional scaling analysis of human and mouse proximal NDRs shows that dimension 1 (Dim.1) mainly separates these two species, while dimension 2 (Dim.2) reflects the major differences between FGCs and somatic cells, together demonstrating both species specificity and functional significance of the chromatin states of proximal NDRs for mammalian germ cell development (Figure 5E and Supplementary information, Figure S6E).

**Figure 6:**
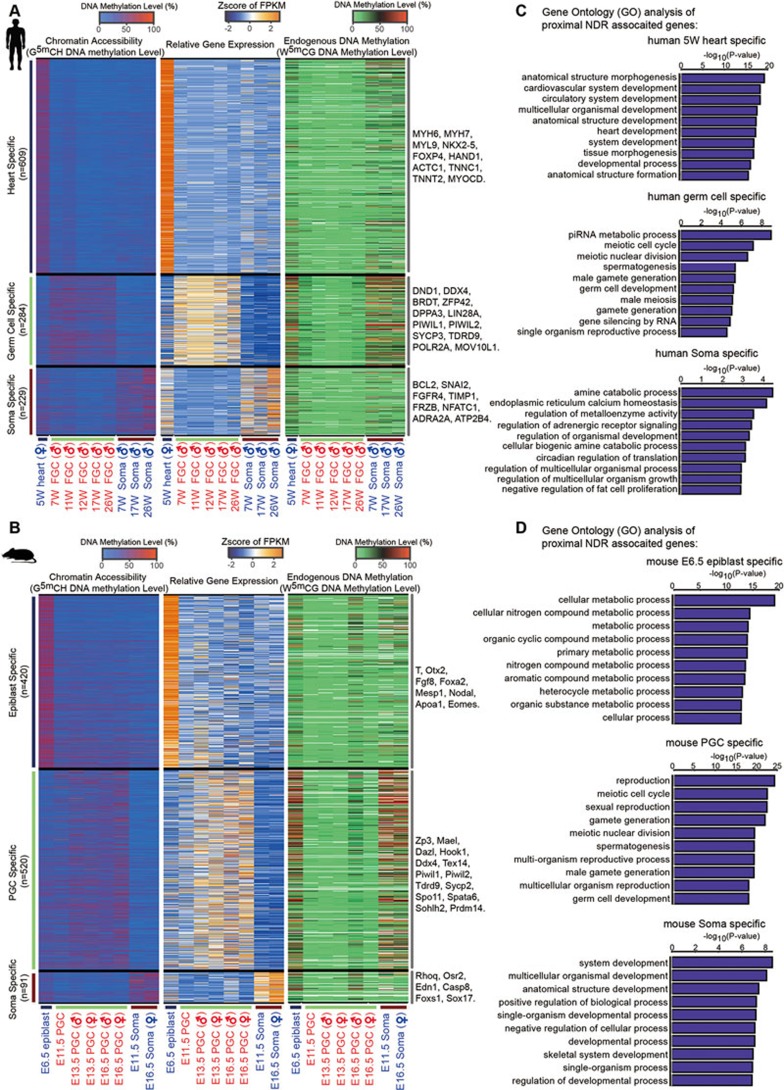
Tissue-specific proximal NDR identification. **(A**, **B)** Heatmaps showing tissue-specific proximal NDRs and the associated genes in human **(A)** and mouse **(B)** samples. **(C**, **D)** Gene ontology (GO) analysis of tissue-specific proximal NDR-associated genes in human **(C)** and mouse **(D)** samples.

We subsequently identified several hundreds of genes with the accessibility of the promoter regions most positively correlated with their expression levels, and further categorized them into heart (or epiblast in mouse)-specific, FGC-specific and gonadal somatic cell-specific proximal NDR-associated genes. As shown in Figure 6A and 6B, cell type-specific proximal NDR-associated genes tend to have more open promoters and higher expression levels than those in the remaining cell types. Human heart-specific proximal NDR-associated genes, such as *MYL9*, *NKX2-5*, *FOXP4*, *MYH6*, *ACTC1*, *TNNT2* and *HAND1*, are significantly enriched for terms of cardiovascular system or heart development in our gene ontology (GO) analysis (Materials and Methods), whereas human FGC-specific proximal NDR-associated genes, such as *SYCP3*, *TDRD9*, *DDX4*, *PIWIL2*, *BRDT* and *DND1*, show a strong enrichment for GO terms of piRNA metabolic process, meiotic cell cycle or spermatogenesis (Figure 6C). Similarly, mouse epiblast-specific proximal NDR-associated genes are preferentially enriched for GO terms of cellular metabolic processes, whereas mouse PGC-specific proximal NDR-associated genes are enriched for reproduction, meiotic cell cycle and gamete generation-related terms (Figure 6D). These findings suggest that the accessibility of the promoter regions of cell fate determination genes contribute to the precise spatial-temporal regulation of their expression.

### Chromatin accessibility at annotated genomic elements and repetitive elements

**Figure 7:**
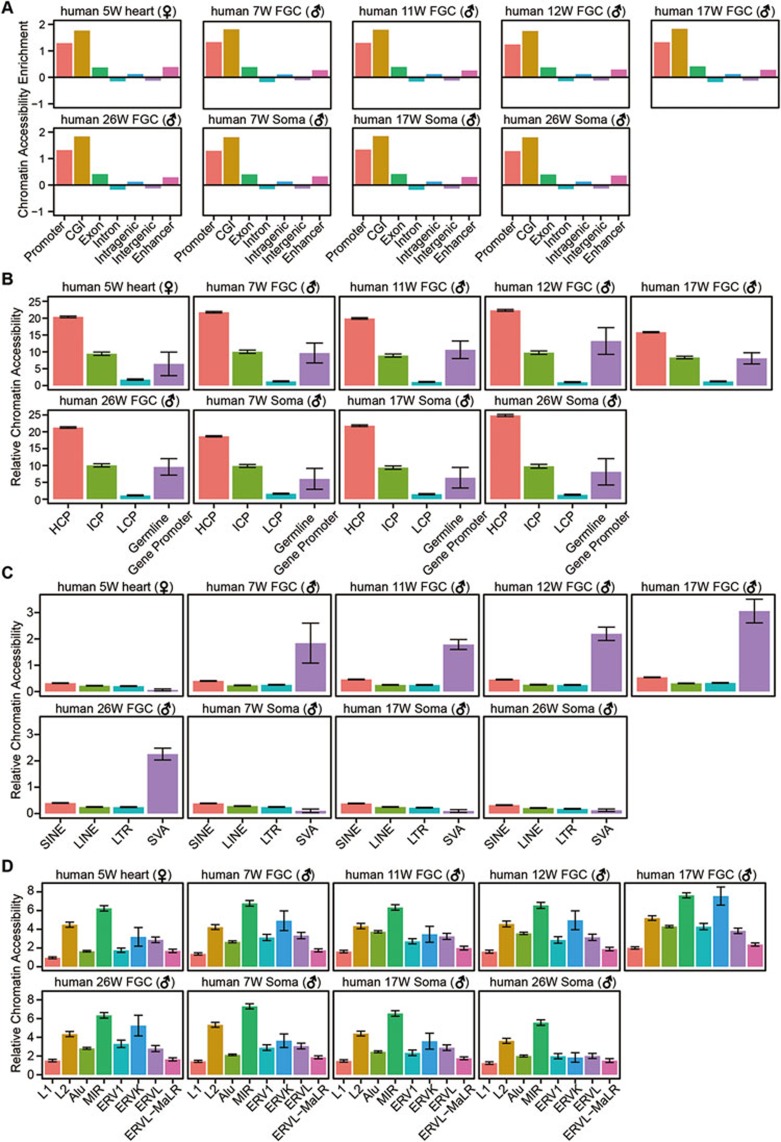
Chromatin accessibility at annotated elements and repetitive elements in human. **(A)** The relative enrichment analysis of chromatin accessibility at the genomic regions. **(B)** Chromatin accessibility of high-density CpG promoter (HCP), intermediate-density CpG promoter (ICP), low-density CpG promoter (LCP) and promoter of germline-specific genes in human germline. **(C**, **D)** Chromatin accessibility at the repetitive elements **(C)** and their subfamilies **(D)** with different evolutionary ages in human germline.

Next, we explored the genome-wide distribution of accessible chromatin with respect to various genomic elements. We found that CpG islands (CGIs) and promoters are the most highly accessible regions. Intragenic regions are more accessible than intergenic regions, with exons more accessible than introns, and enhancers are also open as expected. These patterns are consistent in FGCs and somatic cells in both human and mouse (Figure 7A and Supplementary information, Figure S7A). Further classification of promoters into high-density CpG promoters (HCPs), intermediate-density CpG promoters (ICPs) and low-density CpG promoters (LCPs) shows that HCPs are more open than ICPs, and LCPs have relatively lower accessibility; and genes with HCP tend to have the highest expression level, whereas genes with LCP are expressed at relatively low levels (Figure 7B and Supplementary information, Figure S7B).

When we examined the repeat elements, we found that SINE/variable number of tandem repeats/Alu elements (SVAs) are specifically open in human germline, and also have significantly higher accessibility than SINEs, LINEs and long-terminal repeats (LTRs) (Figure 7C). SVAs also have the most abundant transcripts in human FGCs compared with neighboring somatic cells (Supplementary information, Figure S8A). In mouse, SINEs and LTRs are more accessible than LINEs, and intracisternal A particles (IAPs) are more likely to be open in mouse PGCs than in somatic cells and epiblast (Supplementary information, Figure S7C).

Furthermore, when we categorized the repetitive elements into subfamilies according to their evolutionary ages, we found a more interesting pattern. Both LINE-1 (L1) and LINE-2 (L2) belong to LINE family, with L1 evolutionarily younger than L2. And both Alu and MIR belong to SINE family, with Alu evolutionarily younger than MIR. We found that evolutionarily younger L1 is less open than L2, and evolutionarily younger Alu is less accessible than MIR in human FGCs (Figure 7D and Supplementary information, Figure S7D). In addition, the evolutionarily younger subfamilies, L1 and Alu, tend to have more active transcription activities than the evolutionarily older ones, and also retain higher levels of residual DNA methylation than the older ones after the global DNA demethylation in human and mouse FGCs, consistent with our previous reports^5,33,34^ (Supplementary information, Figure S8). Taken together, these findings indicate that during the DNA methylation reprogramming process, mammalian FGCs tend to retain more residual DNA methylation and adopt a less accessible chromatin state in the evolutionarily younger and more hazardous transposable elements to, potentially, repress their transcription and transposition.

### The relationship between histone modification and chromatin accessibility

Accompanying global reprogramming of the endogenous DNA methylation, the histone modifications in mammalian FGCs were also reported to be reprogrammed genome-wide^8,9,10^. We analyzed the relationship between histone modifications and chromatin accessibility in mammalian germ cells at fine resolution. We incorporated the previously published histone ChIP-seq data set in mouse PGCs^10^, and called H3K4me3, H3K27me3 and H3K27ac peaks independently using our previously reported ChIP-seq analysis pipeline^35^. We find that the endogenous DNA methylation levels of the genomic regions within H3K27ac, H3K4me3 and H3K27me3 peaks and 1 kb upstream/downstream flanking regions are very low in E11.5 and E13.5 mouse PGCs, which is consistent with the global hypomethylation patterns in E11.5 and E13.5 PGCs (Supplementary information, Figure S9A), while from the GCH DNA methylation levels in E11.5 and E13.5 PGCs, we can see that the H3K4me3-marked regions are more accessible than H3K27me3-marked regions at these developmental stages. When we systematically compared signal intensities of the ChIP peaks with chromatin accessibility or endogenous DNA methylation levels of the genome using non-overlapped 1 kb bins, we found that, in general, H3K4me3, H3K27ac and H3K27me3 histone modifications are clearly correlated with chromatin accessibility, with H3K4me3 having the highest correlation with chromatin accessibility than H3K27ac, and H3K27me3 being marginally correlated with chromatin accessibility. These data collectively suggest that chromatin regions with H3K4me3 modification are most likely in open states in mouse PGCs. Notably, we also found anti-correlation relation between the endogenous DNA methylation levels and H3K4me3/H3K27ac/H3K27me3 ChIP-seq signals as expected (Supplementary information, Figure S9B and S9C).

### The relationship between DNA hydroxymethylation and chromatin accessibility

We further integrated the previously published TAB-seq data of human FGCs from 10-week male embryo^5^, and found that promoter and CGI regions tend to have relatively higher 5 hmC levels, and also have higher chromatin accessibility in our NOMe-seq data set (human FGCs from 11-week embryo), suggesting that open chromatins probably undergo active demethylation (Supplementary information, Figure S10A and S10B).

Furthermore, when we grouped RefSeq genes into 10 deciles according to the DNA hydroxymethylation level of their promoter regions (Supplementary information, Figure S10C), we found that the genes with intermediate hydroxymethylation levels are more likely to have higher chromatin accessibility and also have higher expression levels. In addition, when we divided the genes into 10 deciles based on the DNA hydroxymethylation levels of the gene body regions, we found that genes with intermediate levels of hydroxymethylation in gene bodies tend to have higher expression levels (Supplementary information, Figure S10D).

Besides the accessible chromatin regions, we found that the nucleosome signals strongly peak at the exon-intron boundaries of the RefSeq genes, and are more preferentially positioned on exons relative to introns in human and mouse FGCs, whereas nucleosomes tend to occupy weakly at these boundaries in human and mouse gonadal somatic cells (Supplementary information, Figure S11A and S11B). Consistent with previous reports, this finding indicates that nucleosomes have potential roles in exon-intron splicing and possibly regulate the RNA polymerase processivity^36,37,38^, and this mechanism is probably enhanced in the FGCs when the global DNA methylation is erased.

## Discussion

To date, several genome-wide chromatin accessibility profiling techniques have been developed to map and characterize open chromatin regions and nucleosome patterning in cell or tissue samples. Some of the techniques, such as transposase-accessible chromatin with high-throughput sequencing (ATAC-seq) or DNase-seq, have already been optimized to capture chromatin organization from small amount of cells or even at single-cell resolution, refining our understanding of the epigenetic regulation of cell identity to a greater extent^39,40,41,42^. But both the ATAC-seq and DNase-seq techniques rely on the abilities and processivities of Tn5 transposase or DNase enzyme, which can preferentially insert into or digest the accessible chromatin in the genome, leaving the closed chromatin regions and undetected regions indistinguishable. NOMe-seq is built on *ex vivo* methyltransferase activity of the M.CviPI enzyme, which can artificially methylate the cytosine of the GpC sites in open chromatin regions, but keep the cytosine of the GpC sites in 'closed' chromatin regions unmethylated. This allows the open chromatin regions, closed chromatin regions and undetected chromatin regions to be discriminated. Moreover, it also gives readouts of the endogenous CpG DNA methylation within the same DNA molecule. To our knowledge, this is the first time that NOMe-seq is applied to mammalian germ cells and our data provide a comprehensive and in-depth picture of the dynamics of chromatin accessibility and DNA methylation within the same cells.

**Figure 8:**
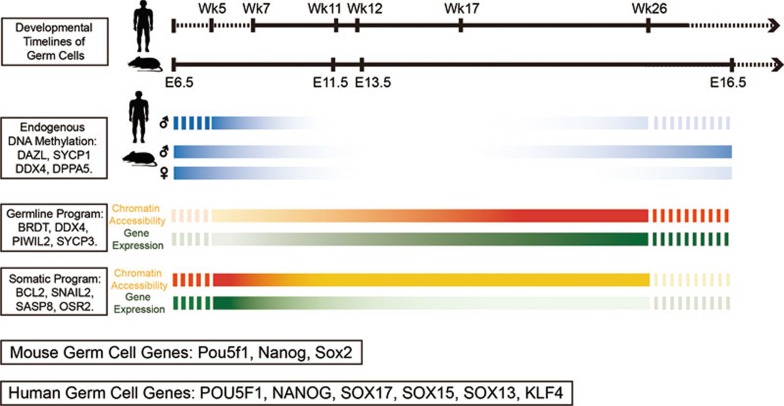
Sketch of reprogramming dynamics of endogenous DNA methylation, chromatin accessibility, as well as gene expression patterns during mouse and human germ cell development.

The general patterns of chromatin accessibility and DNA methylation in human FGCs we show here are similar to those in mouse at the comparable developmental stages; and both human and mouse FGCs undergo genome-wide DNA demethylation to achieve proper reprogramming and removal of the parental epigenetic memory. Chromatin of the proximal NDRs and distal NDRs of the germline-specific genes or some germline-specific retroelements tends to be open in both human and mouse FGCs, and expression of the corresponding genes is upregulated as the consequence, suggesting evolutionary conservation of reprogramming of the epigenome and functional modulation of chromatin accessibility during FGC development *in vivo* (Figure 8).

Distal NDRs of human and mouse FGCs are preferentially enriched for binding motifs of germline-specific TFs, such as OCT4, NANOG, SOX2/SOX17 and PRDM14; and also some other critical chromatin structure organizers, such as CTCF and BORIS, suggesting that chromatin state of these distal elements (some are potential germline-specific enhancers) is likely to be an major determinant of cell identity, especially when the genome is free of any endogenous DNA methylation.

Another interesting finding that emerged from our study is that SVAs are specifically open in the human FGCs, but not in the neighboring somatic cells; and SVAs have the most abundant transcripts in human FGCs compared with the neighboring somatic cells. Our high-resolution chromatin accessibility map of repetitive elements should provide insights to the potential function of chromatin state in regulation of expression of the repetitive elements in human FGCs.

Furthermore, we found that the chromatin states of evolutionarily younger subfamilies of repeat elements such as L1 of LINE family and Alu of SINE family tend to be less accessible than their evolutionarily older counterparts in human FGCs. Together with our previous work^5^, these findings suggest that during the global DNA demethylation, human FGCs tend to maintain more residual DNA methylation and a less accessible chromatin state in the evolutionarily younger and probably more active and deleterious transposable elements to repress their transcription and transposition.

Finally, the overall landscapes of chromatin accessibility and DNA methylation in mouse and human FGCs reinforce the view that both epigenetic mechanisms are globally reprogrammed and, in the midst of these tremendous reorganizations, work nevertheless synergistically to support the proper development of mammalian germline.

## Materials and Methods

### Informed consent and ethics approval

This study was approved by the Reproductive Study Ethics Committee of Peking University Third Hospital (Research license number 2012SZ015). The aborted human embryos used for human FGCs collection in this study were obtained with fully informed patient consent. Human embryos used in this study are staged by the developmental weeks, which were counted from the speculated moment of fertilization^43^. And all mouse material collection was authorized by Peking University and performed according to the procedures described elsewhere^44^.

### Human and mouse sample preparation

The de-identified fetus at 7-26 weeks of gestation was dissected under the microscopes, and the gonad was extensively washed with DPBS to remove any blood and other contaminants, then digested with 500 μl of Accutase Cell Detachment Solution (Millipore #SCR005) for 10-25 min at 37 °C. After digestion, the single-cell suspension was obtained by filtering through 30 μm Pre-Separation Filters (MiltenyiBiotec #130-041-407) before centrifuging at 300× *g* for 10 min at 4 °C. The cell pellet was washed with DPBS and finally resuspended with 300 μl L15 medium (Gibco # 21083027) with 10% FBS. Accordingly to the previous publication^45^, we chose CD117 (also known as KIT) surface marker to isolate KIT-positive FGCs. Briefly, 100 μl of FcR Blocking Reagent and 100 μl of CD117 MicroBeads (MiltenyiBiotec #130-091-332) were added to the 300 μl gonad cell suspension and mixed well by gently pipetting. Then, 10 μl of PE Mouse Anti-Human CD117 antibody (BD Pharmingen #555714, clone YB5.B8) was added and mixed well before incubation at 4 °C for 30 min. After centrifuge at 300× *g* for 10 min at 4 °C, the cell pellet was resuspended in 500 μl L15 medium with 10% FBS, and then subjected to the MACS (MiltenyiBiotec). After MACS enrichment, the KIT-positive fraction was further sorted by FACS (BD FACSAriaII). All the human FGCs used in this study were sorted by MACS coupled with FACS, which are restricted to the KIT-positive population of the germ cells, and all the somatic cells were collected as the KIT-negative fraction after FACS. Human heart tissue was obtained from 5-week fetus with careful dissection, and all the blood was squeezed out and heart was further washed several times with DPBS before digestion with Accutase Cell Detachment Solution for 15 min at 37 °C.

Mouse E11.5, E13.5 and E16.5 PGCs were isolated from timed mated females carrying the Oct4-Gfp transgene expressed in the developing gonad with CD-1 background. For each stage, 10-30 embryos were dissected and gonads were pooled, and E13.5 and E16.5 male and female embryos were distinguished morphologically and collected separately. The pooled gonads were digested with 500 μl of Accutase Cell Detachment Solution (Millipore #SCR005) for 10-20 min at 37 °C, and the digestion reaction was quenched after adding 500 μl L15 medium (Gibco # 21083027) with 10% FBS, the cells were filtered through 30 μm Pre-Separation Filters (MiltenyiBiotec #130-041-407) and then pelleted by centrifuging at 300× *g* for 10 min at 4 °C. The mouse PGCs at each time point were collected after BD FACSAriaII cell sorter as GFP-positive fraction, the gonadal somatic cells were collected as the GFP-negative fraction. Mouse epiblasts were obtained at E6.5 with manually dissected under microscopy, and further separated away with the extraembryonic tissues. And single-cell suspension of mouse epiblasts was obtained after digestion with Accutase Cell Detachment Solution for 10 min at 37 °C.

All the cells isolated from the FACS cell sorter or just digested with Accutase solution were further washed with DPBS, pelleted and freezed at −80 °C for temporary storage.

### Nucleosome occupancy and methylome sequencing

Approximately 1 000-10 000 cells were used for the NOMe-seq in this study. Briefly, cell pellet was first lysed in 1× lysis buffer (part of the NOMe-Seq Kit, Active motif #54000) with Protease inhibitor and PMSF (part of the NOMe-Seq Kit, Active motif #54000) on ice for 1 h with intermittent vortex. The nuclei was released, washed with DPBS, and spiked in with 0.1% unmodified lambda DNA (ThermoFisher #SD0011), and then subjected to the 15 U M.CviPI GpC Methyltransferase (NEB #M0227L) treatment for 1 h at 37 °C, supplemented with 160 nM fresh SAM (NEB #B9003S), then followed by a boost with an additional 15 U M.CviPI and 160 nM fresh SAM for another 2 h at 37 °C. The reaction was stopped by adding EDTA followed by Proteinase K digestion at 55 °C overnight, then genomic DNA was column-purified using Genomic DNA Clean and Concentrator Kit (VisTech #DC2008). For NOMe-seq, the libraries were prepared using PBAT protocol with some minor modifications^46,47^. Briefly, the isolated genomic DNA was first bisulfite converted using MethylCode Bisulfite Conversion Kit (ThermoFisher #MECOV-50), and first strand was synthesized using random nonamer primers with biotin-tagged truncated Illumina P5 adapter (5′-biotin-CTACACGACGCTCTTCCGATCTNNNNNNNNN-3′), and second strands were synthesized using random nonamer primers containing a truncated P7 Illumina adapter (5′-AGACGTGTGCTCTTCCGATCTNNNNNNNNN-3′), the final library was amplified with 4-6 cycles of PCR using 1 U Kapa HiFi HS DNA Polymerase (KAPA Biosystems), and then AMpure XP Beads (Beckman Coulter) purified, quantified and pooled on Illumina HiSeq 2500 platform with 100 or 150 bp paired-end mode (sequenced by Novogene).

### RNA-seq library preparation and sequencing

Total RNAs of human 5-week heart, mouse E6.5 epiblasts, mouse PGCs and corresponding somatic cells at different stages are isolated and RNA from these cells are extracted using RNeasy mini kit (Qiagen), and bulk RNA-seq libraries are constructed under the instruction of NEBNext Ultra RNA Library Prep Kit for Illumina (NEB).

For other human samples, including KIT-positive FGCs and KIT-negative gonadal somatic cells at different development stages, we first picked the single cells using mouth pipette after FACS sorting, and obtained the single-cell cDNA library using the single-cell RNA-seq protocol we developed in 2009^48,49^. The amplified cDNAs were fragmented into 200-300 bp using Covaris S2 system, and the final single-cell RNA-seq library was prepared using NEBNext Ultra DNA Library Prep Kit for Illumina (NEB).

The quality-ensured RNA-seq libraries were also pooled and sequenced on HiSeq 2500 platform with 100 or 150 bp paired-end mode (sequenced by Novogene).

### Data processing and bioinformatic analysis

NOMe-seq data processing

1. Data quality control

Adapter or other artificial sequence polluted reads, such as the illumina sequencing adapters or reads with more than 10% N (undetermined bases), were filtered out prior to the analysis using customized Perl scripts.

2. Sequencing alignment

Cleaned reads were mapped to the mouse or human reference genomes (mm9 or hg19 version) following our modified bisulfite sequencing pipeline^5^. PCR duplicates are removed using SAMtools^50^, and the DNA methylation level of every cytosine was calculated according our previous publications^33,51^. According to previous reports, GCG and CCG context should be excluded in the subsequent analysis^19^. Because cytosine methylation can be designated as endogenous CpG methylation or artificial M.CviPI GpC methyltransferase treatment within GCG trinucleotides, while CCG methylation can be attributed to the slight 'off-target' M.CviPI GpC methyltransferase activity. That is to say, we only focus on ACG/TCG trinucleotides (WCG, W indicates A and T nucleotides) to estimate the endogenous CpG methylation level, and GCA/GCT/GCC (GCH, H represents A, T and C nucleotides) to deduce the nucleosome position and chromatin accessibility.

3. Data reproducibility

In each human and mouse stage, we performed 2-3 replicates if materials available. The WCG and GCH methylation levels along the transcripts and their flanking regions are highly reproducible across replicates. Also, we spiked in 5% unmethylated lambda DNA to estimate the *in vitro* methylation efficiency of the M.CviPI, also the non-conversion rate during the bisulfite treatment. Only the samples with no less than 90% *in vitro* methylation efficiency, and also higher than 98% bisulfite conversion rate were retained for the downstream analysis.

4. Nucleosome-depleted regions and nucleosome position identification

Only the DNA methylation information in GCH sites was used to call NDR and nucleosome. First, the genome was sized to 100 bp sliding windows with 20 bp steps, and the C and T read counts of every GCH site with no less than 3 depths in each window were summed up and *p*-values (*χ*^2^-test) for the enrichment of unmethylated GCH sites of each window were calculated as the differences to the genome background. Only the significant windows with *p*-values passed the cutoffs (−log10(*p*-value) > 5) also with a minimum size of 140 bp were retained for the downstream analysis. And the NDRs were further classified as the proximal NDRs and distal NDRs depending on their distances between NDR centers and the transcriptional start sites.

Similarly, the nucleosome position was identified by using the similar strategy with 40 bp sliding windows, 20 bp steps. Only windows with *p*-values (*χ*^2^-test) for the enrichment of methylated GCH sites passed the cutoffs (−log10(*p*-value) > 3) and with a minimum size of 60 bp were kept for downstream analysis.

5. NDR-enriched motifs analysis

HOMER (version 4.7.2) was applied to identify the binding motifs and further calculate the enrichment scores for different TFs for TSS NDRs and distal NDRs with the following command:

findMotifsGenome.pl input.bed hg19 (or mm9) output_dir -size 2000 -len 8 -S 100^35,52^.

6. Evolutionary comparison between human and mouse

In order to compare the endogenous DNA methylation information (WCG methylation level) and the chromatin accessibility (GCH methylation level) between human and mouse species with minimal biases, the mouse genome coordinate (mm9) was lifted over to the human assembly (hg19) using UCSC LiftOver tool (https://genome.ucsc.edu/cgi-bin/hgLiftOver), together with the genomic annotations. The endogenous DNA methylome and chromatin accessibility of mouse samples were re-analyzed using the mouse-to-human homology DNA segments, and spearman coefficients were computed and hierarchical clustering was performed using the 'hclust' function, and 'ward.D2' method with 'pairwise.complete.obs' in R software. PCA was performed using pca and prcomp package in Bioconductor^53,54^.

To ensure proper transcriptome comparison between these two species, only the 16 391 well-annotated human-mouse orthologous genes downloaded from the Vertebrate Homology Database (http://www.informatics.jax.org/homology.shtml) were used for further analysis.

7. Stage-specific proximal NDR identification

Both the endogenous DNA methylation level (WCG DNA methylation level) of the promoter regions and the chromatin accessibility (GCH DNA methylation level) of these regions contribute to the regulation of corresponding gene expression level. To identify the stage-specific (or cell type-specific) proximal NDRs and also their related genes, we systematically computed the spearman correlation coefficients of endogenous DNA methylome, chromatin accessibilities and gene expression levels, and only the proximal NDRs with correlation higher than 0.6 and *p*-values < 0.1 were retained and further classified into heart (or epiblast), FGC and somatic cell-specific proximal NDRs, respectively. GO analysis of these proximal NDR-related genes was performed using R GOstats package^55^.

### RNA-seq data processing

For the RNA-seq data, the cleaned reads were aligned to the hg19 or mm9 reference genome using TopHat (version 2.0.9) with the default parameters^56^. And the transcript annotations in GTF format downloaded from the genecode project (http://www.gencodegenes.org) with the version v19 for human and vM1 for mouse. The regions for protein-coding genes and lncRNAs were also defined in the GTFs. The gene expression levels (FPKM) of each sample were calculated using the cuffinks^57^.

### Data accession

All the NOMe-seq and RNA-seq raw data and processed data were deposited to the Gene Expression Omnibus (GEO) database with the accession number: GSE79552.

## Author Contributions

FT and JQ conceived the project. HG, LY, JY, YW, YG, FG, YH, XF, XW, XZ, JY, YW, HJ, WZ and LW performed the experiments. BH, JD and HG conducted the bioinformatics analyses. FT, JQ and HG wrote the manuscript with help from all the authors.

## Competing Financial Interests

The authors declare no competing financial interests.

## Supplementary Information

Supplementary information, Table S1 The sequencing statistics of the NOMe-seq datasets in human and mouse.

Supplementary information, Table S2 The number of distal and proximal NDRs identified in human and mouse samples.

Supplementary information, Table S3 The tissue specific proximal NDR identification and the associated genes in human and mouse samples.

Supplementary information, Figure S1 Reproducibility of the NOMe-seq data of mouse germline.

Supplementary information, Figure S2 Reproducibility of the NOMe-seq data of human germline.

Supplementary information, Figure S3 The *in vitro* methylation efficiency of M.CviPI GpC methyltransferase and bisulfite conversion rate of the NOMe-seq.

Supplementary information, Figure S4 The endogenous DNA methylation and chromatin accessibility dynamics of the sex chromosome in mammalian germ cells.

Supplementary information, Figure S5 The relationships among chromatin accessibility, endogenous DNA methylation and gene expression of single copy gene.

Supplementary information, Figure S6 The clustering analysis of accessibility of the NDRs in mammalian PGCs.

Supplementary information, Figure S7 Chromatin accessibility at annotated elements and repetitive elements in mouse.

Supplementary information, Figure S8 The relationships among chromatin accessibility, endogenous DNA methylation and gene expression of repetitive elements.

Supplementary information, Figure S9 The relationships between the histone modifications and the chromatin accessibilities in mouse PGCs.

Supplementary information, Figure S10 The relationships between the DNA hydroxymethylation and the chromatin accessibility in human fetal germ cells.

Supplementary information, Figure S11 The nucleosome patterning on the intron-exon boundary.
