# Supplementary material for: DNA methylation and chromatin accessibility profiling of mouse and human fetal germ cells
Source: Cell Res. 2016 Nov 8;27(2):165–83. doi: 10.1038/cr.2016.128 (PMC5339845; doi:10.1038/cr.2016.128)
Supplement: Supplementary information, Figure S1 — Reproducibility of the NOMe-seq data of mouse germline. [file cr2016128x4.pdf]

Figure S1

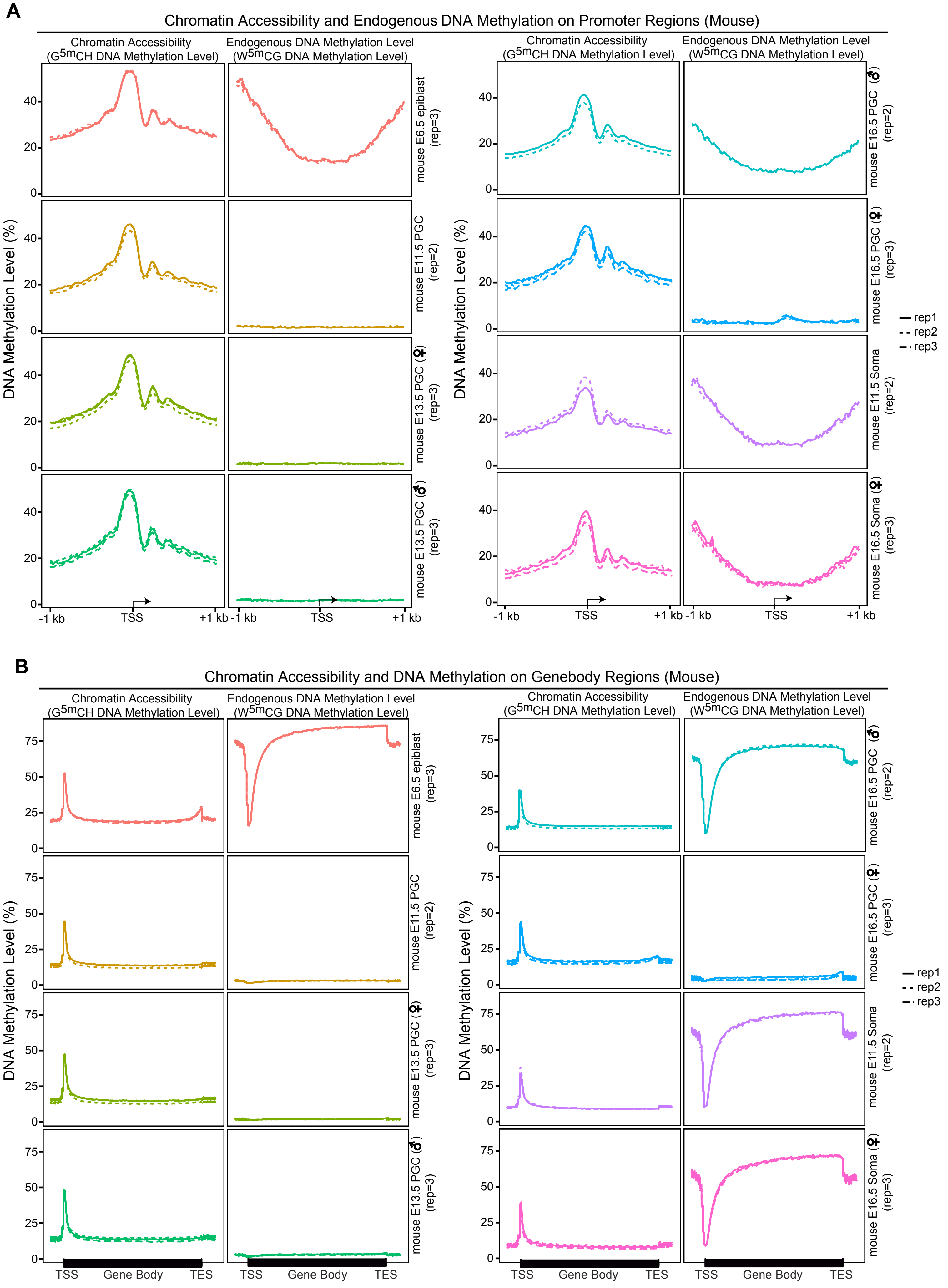

A
Chromatin Accessibility and Endogenous DNA Methylation on Promoter Regions (Mouse)
Chromatin Accessibility (G5mCH DNA Methylation Level)
Endogenous DNA Methylation Level (W5mCG DNA Methylation Level)
DNA Methylation Level (%)
mouse E6.5 epiblast (rep=3)
mouse E11.5 PGC (rep=2)
mouse E13.5 PGC (♀) (rep=3)
mouse E13.5 PGC (♂) (rep=3)
mouse E16.5 PGC (♂) (rep=2)
mouse E16.5 PGC (♀) (rep=3)
mouse E11.5 Soma (rep=2)
mouse E16.5 Soma (♀) (rep=3)
-1 kb
TSS
+1 kb
rep1
rep2
rep3
B
Chromatin Accessibility and DNA Methylation on Genebody Regions (Mouse)
Gene Body
TES

Figure S1 Reproducibility of the NOMe-seq data of mouse germline.
(A) The reproducible patterns of chromatin accessibility and endogenous DNA methylation on promoter regions across replicates. (B) The reproducible patterns of chromatin accessibility and endogenous DNA methylation on gene body regions across replicates.
