# Supplementary material for: DNA methylation and chromatin accessibility profiling of mouse and human fetal germ cells
Source: Cell Res. 2016 Nov 8;27(2):165–83. doi: 10.1038/cr.2016.128 (PMC5339845; doi:10.1038/cr.2016.128)
Supplement: Supplementary information, Figure S2 — Reproducibility of the NOMe-seq data of human germline. [file cr2016128x5.pdf]

Figure S2

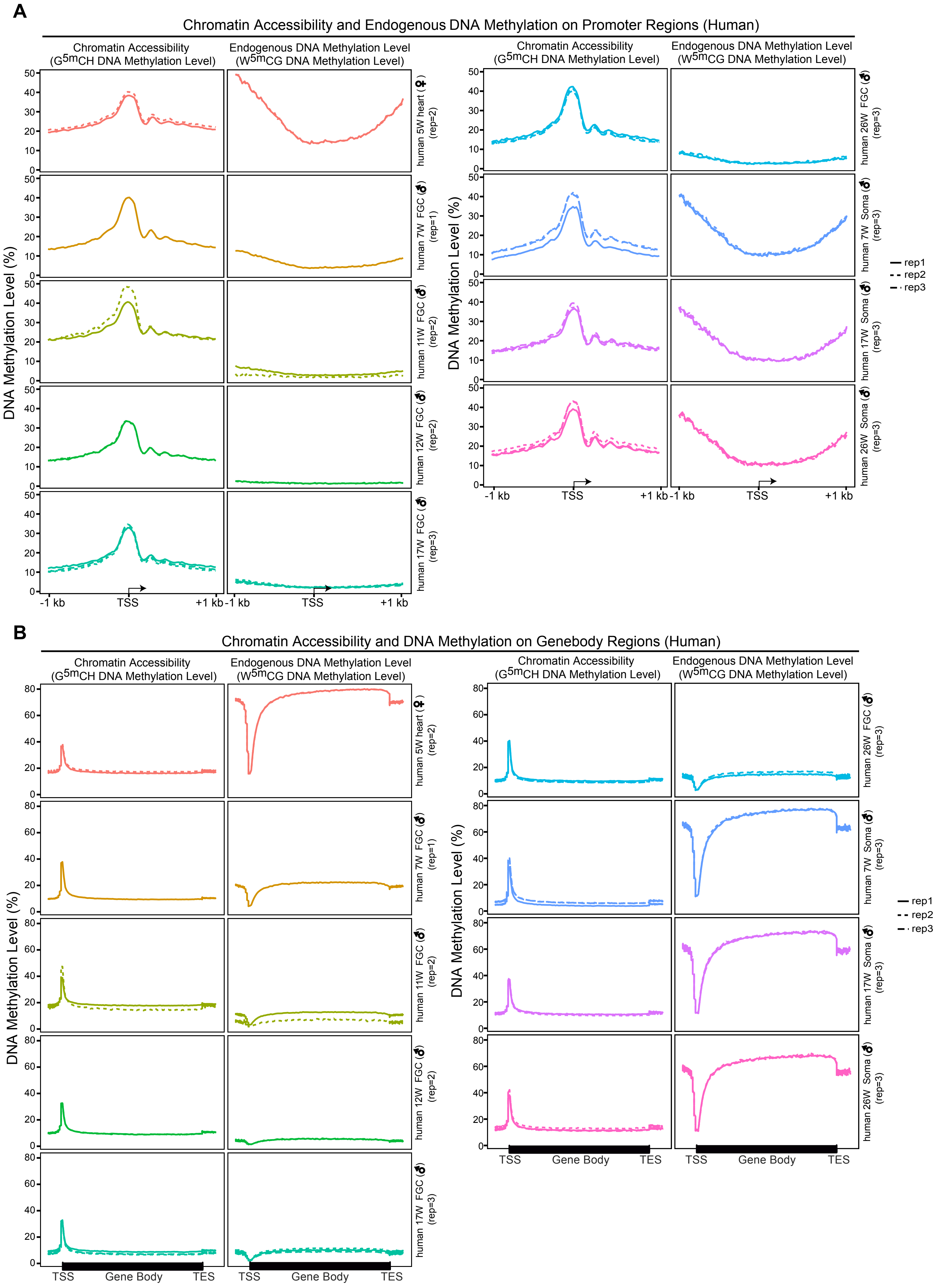
A
Chromatin Accessibility and Endogenous DNA Methylation on Promoter Regions (Human)
Chromatin Accessibility (G5mCH DNA Methylation Level)
Endogenous DNA Methylation Level (W5mCG DNA Methylation Level)
DNA Methylation Level (%)
human 5W heart (♀) (rep=2)
human 7W FGC (♂) (rep=1)
human 11W FGC (♂) (rep=2)
human 12W FGC (♂) (rep=2)
human 17W FGC (♂) (rep=3)
human 26W FGC (♂) (rep=3)
human 7W Soma (♂) (rep=3)
human 17W Soma (♂) (rep=3)
human 26W Soma (♂) (rep=3)
rep1
rep2
rep3
-1 kb
TSS
+1 kb
B
Chromatin Accessibility and DNA Methylation on Genebody Regions (Human)
TSS
Gene Body
TES

Figure S2 Reproducibility of the NOMe-seq data of human germline.
(A) The reproducible patterns of chromatin accessibility and endogenous DNA methylation on promoter regions across replicates. (B) The reproducible patterns of chromatin accessibility and endogenous DNA methylation on gene body regions across replicates.
