# Supplementary material for: DNA methylation and chromatin accessibility profiling of mouse and human fetal germ cells
Source: Cell Res. 2016 Nov 8;27(2):165–83. doi: 10.1038/cr.2016.128 (PMC5339845; doi:10.1038/cr.2016.128)
Supplement: Supplementary information, Figure S3 — The in vitro methylation efficiency of M.CviPI GpC methyltransferase and bisulfite conversion rate of the NOMe-seq. [file cr2016128x6.pdf]

Figure S3

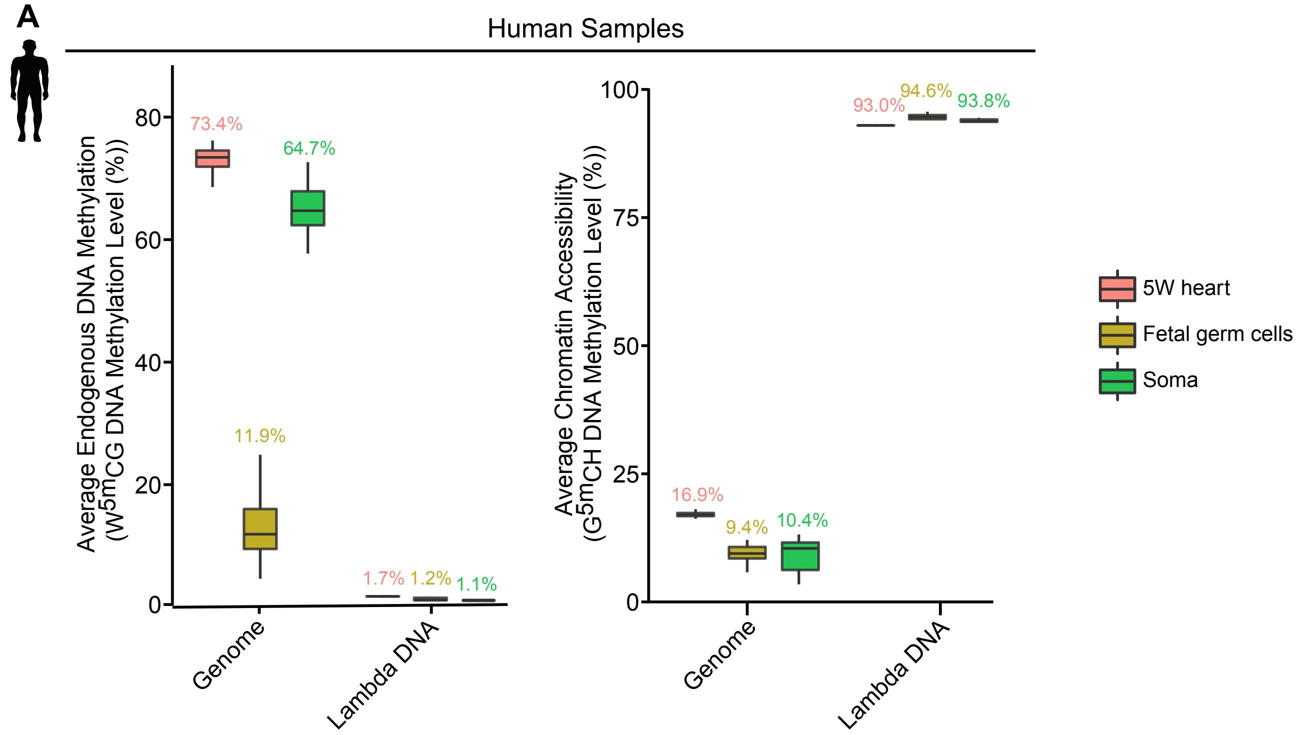
A
Human Samples
Average Endogenous DNA Methylation
(W5mCG DNA Methylation Level (%))
80
60
40
20
0
73.4%
64.7%
11.9%
1.7%
1.2%
1.1%
Genome
Lambda DNA
Average Chromatin Accessibility
(G5mCH DNA Methylation Level (%))
100
75
50
25
0
93.0%
94.6%
93.8%
16.9%
9.4%
10.4%
Genome
Lambda DNA
5W heart
Fetal germ cells
Soma

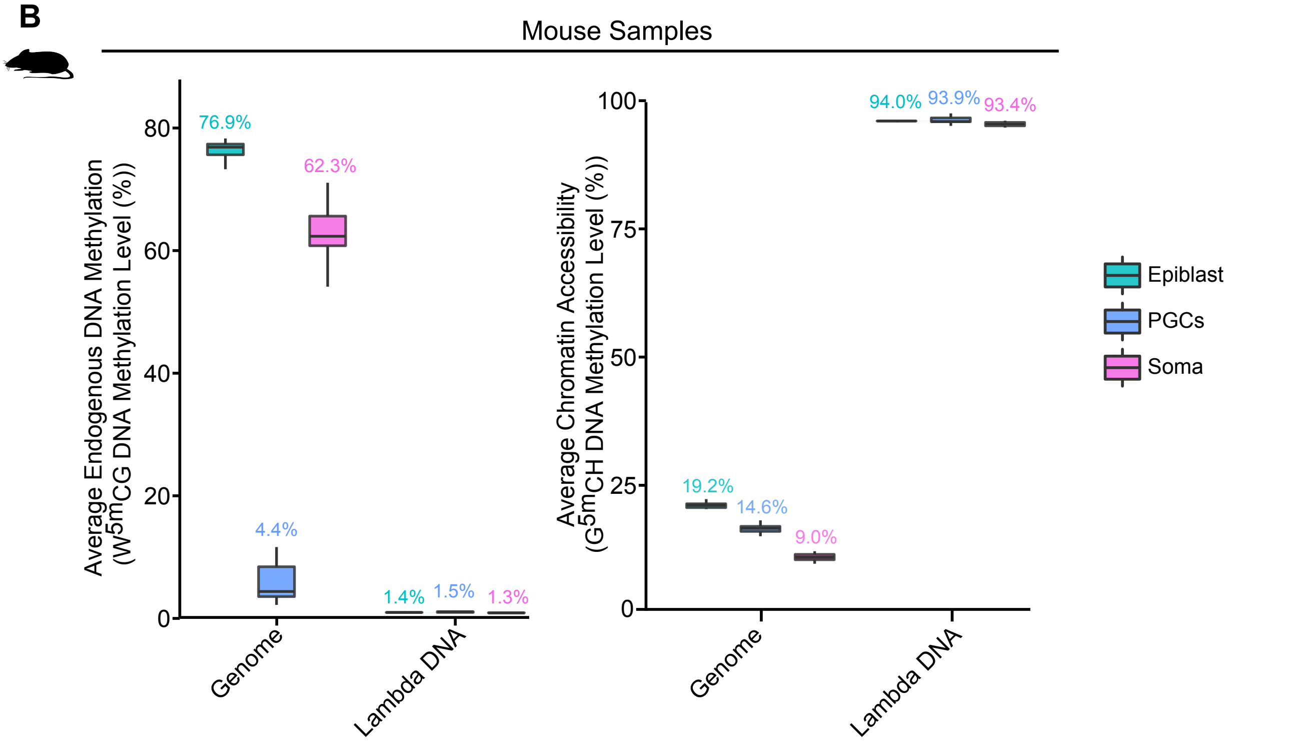
B
Mouse Samples
Average Endogenous DNA Methylation
(W5mCG DNA Methylation Level (%))
80
60
40
20
0
76.9%
62.3%
4.4%
1.4%
1.5%
1.3%
Genome
Lambda DNA
Average Chromatin Accessibility
(G5mCH DNA Methylation Level (%))
100
75
50
25
0
94.0%
93.9%
93.4%
19.2%
14.6%
9.0%
Genome
Lambda DNA
Epiblast
PGCs
Soma

Figure S3 The *in vitro* methylation efficiency of M.CviPI GpC methyltransferase and bisulfite conversion rate of the NOMe-seq.

Both the *in vitro* methylation efficiency of M.CviPI GpC methyltransferase and bisulfite conversion rate are estimated based on the methylation level the GCH sites and WCG sites of the unmethylated lambda DNA spike-ins.
