# Supplementary material for: DNA methylation and chromatin accessibility profiling of mouse and human fetal germ cells
Source: Cell Res. 2016 Nov 8;27(2):165–83. doi: 10.1038/cr.2016.128 (PMC5339845; doi:10.1038/cr.2016.128)
Supplement: Supplementary information, Figure S4 — The endogenous DNA methylation and chromatin accessibility dynamics of the sex chromosome in mammalian germ cells. [file cr2016128x7.pdf]

Figure S4

A

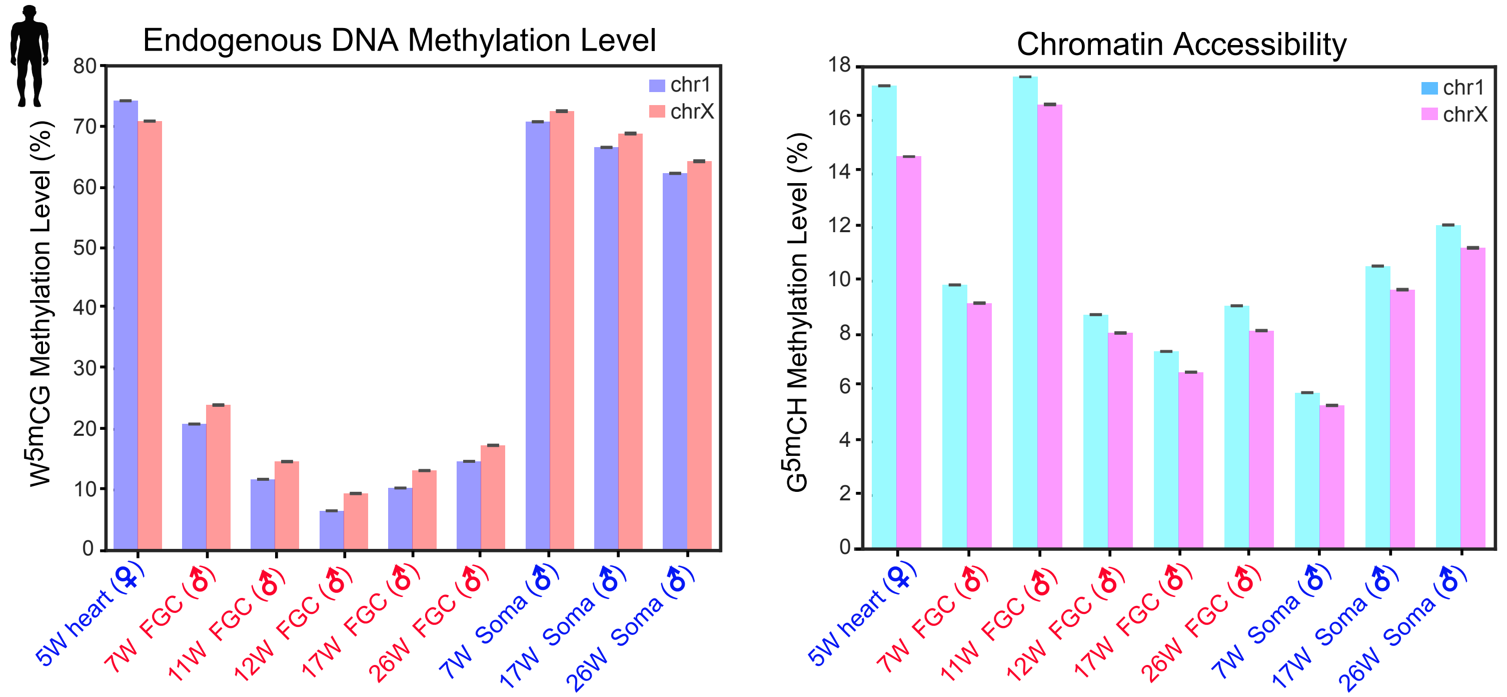
Endogenous DNA Methylation Level
W5mCG Methylation Level (%)
chr1
chrX
0
10
20
30
40
50
60
70
80
5W heart (♀)
7W FGC (♂)
11W FGC (♂)
12W FGC (♂)
17W FGC (♂)
26W FGC (♂)
7W Soma (♂)
17W Soma (♂)
26W Soma (♂)
Chromatin Accessibility
G5mCH Methylation Level (%)
0
2
4
6
8
10
12
14
16
18

B

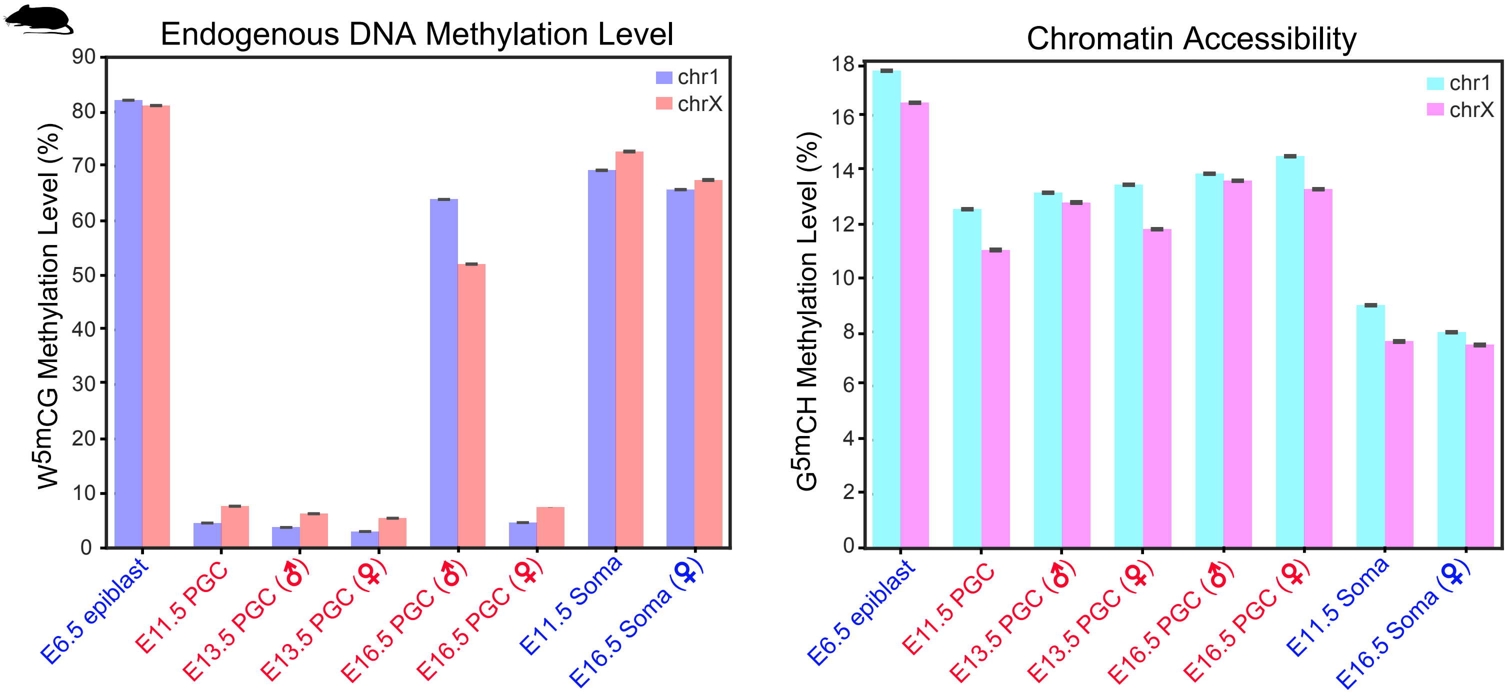
Endogenous DNA Methylation Level
W5mCG Methylation Level (%)
chr1
chrX
0
10
20
30
40
50
60
70
80
90
E6.5 epiblast
E11.5 PGC
E13.5 PGC (♂)
E13.5 PGC (♀)
E16.5 PGC (♂)
E16.5 PGC (♀)
E11.5 Soma
E16.5 Soma (♀)
Chromatin Accessibility
G5mCH Methylation Level (%)
0
2
4
6
8
10
12
14
16
18

Figure S4 The endogenous DNA methylation and chromatin accessibility dynamics of the sex chromosome in mammalian germ cells.

Barplots showing the endogenous DNA methylation and chromatin accessibility dynamics of chromosome X (chrX) and chromosome 1 (chr1) during mammalian germ cell development.
