# Supplementary material for: DNA methylation and chromatin accessibility profiling of mouse and human fetal germ cells
Source: Cell Res. 2016 Nov 8;27(2):165–83. doi: 10.1038/cr.2016.128 (PMC5339845; doi:10.1038/cr.2016.128)
Supplement: Supplementary information, Figure S5 — The relationships among chromatin accessibility, endogenous DNA methylation and gene expression of single copy gene. [file cr2016128x8.pdf]

Figure S5

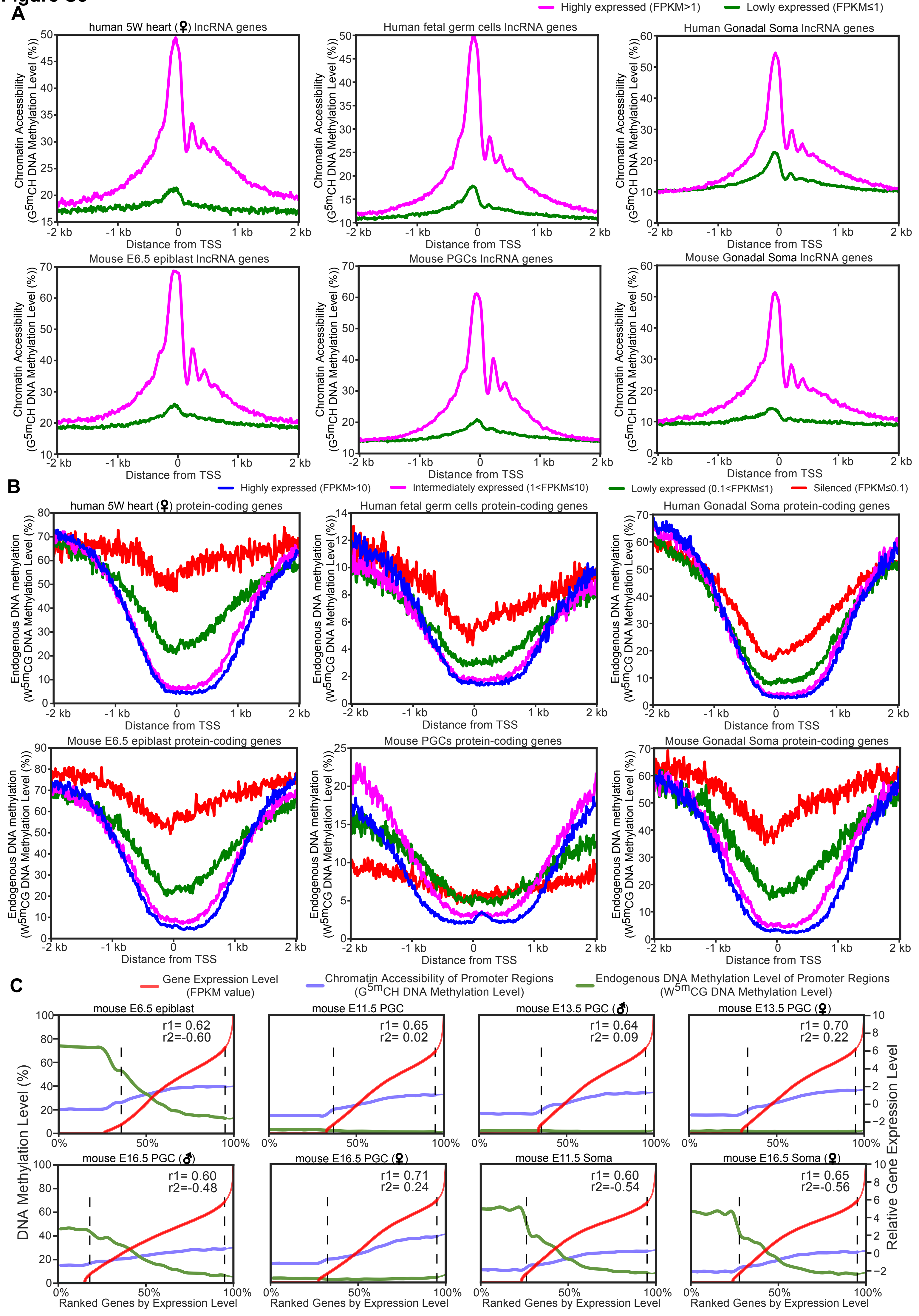
A
Highly expressed (FPKM>1)
Lowly expressed (FPKM≤1)
human 5W heart (♀) lncRNA genes
Human fetal germ cells lncRNA genes
Human Gonadal Soma lncRNA genes
Mouse E6.5 epiblast lncRNA genes
Mouse PGCs lncRNA genes
Mouse Gonadal Soma lncRNA genes
Chromatin Accessibility
(G5mCH DNA Methylation Level (%))
Distance from TSS
-2 kb
-1 kb
0
1 kb
2 kb
B
Highly expressed (FPKM>10)
Intermediately expressed (1<FPKM≤10)
Lowly expressed (0.1<FPKM≤1)
Silenced (FPKM≤0.1)
human 5W heart (♀) protein-coding genes
Human fetal germ cells protein-coding genes
Human Gonadal Soma protein-coding genes
Mouse E6.5 epiblast protein-coding genes
Mouse PGCs protein-coding genes
Mouse Gonadal Soma protein-coding genes
Endogenous DNA methylation
(W5mCG DNA Methylation Level (%))
Distance from TSS
C
Gene Expression Level
(FPKM value)
Chromatin Accessibility of Promoter Regions
(G5mCH DNA Methylation Level)
Endogenous DNA Methylation Level of Promoter Regions
(W5mCG DNA Methylation Level)
mouse E6.5 epiblast
r1= 0.62
r2=-0.60
mouse E11.5 PGC
r1= 0.65
r2= 0.02
mouse E13.5 PGC (♂)
r1= 0.64
r2= 0.09
mouse E13.5 PGC (♀)
r1= 0.70
r2= 0.22
mouse E16.5 PGC (♂)
r1= 0.60
r2=-0.48
mouse E16.5 PGC (♀)
r1= 0.71
r2= 0.24
mouse E11.5 Soma
r1= 0.60
r2=-0.54
mouse E16.5 Soma (♀)
r1= 0.65
r2=-0.56
DNA Methylation Level (%)
Relative Gene Expression Level
Ranked Genes by Expression Level
0%
50%
100%

Figure S5 The relationships among chromatin accessibility, endogenous DNA methylation and gene expression of single copy gene.

(A) The relationship between the chromatin accessibility of the promoter regions and the corresponding lncRNA expression in human and mouse samples. LncRNAs were classified into two groups according to their expression levels. (B) The anti-correlation between the endogenous DNA methylation of the promoter regions and the corresponding RefSeq genes expression in human and mouse samples. (C) The Spearman correlation (r1) between the gene expression level (red) and chromatin accessibility of the promoter regions (lightblue) and the Spearman correlation (r2) between the gene expression level (red) and endogenous DNA methylation level of the promoter regions (green) in mouse PGCs and Soma.
