# Supplementary material for: DNA methylation and chromatin accessibility profiling of mouse and human fetal germ cells
Source: Cell Res. 2016 Nov 8;27(2):165–83. doi: 10.1038/cr.2016.128 (PMC5339845; doi:10.1038/cr.2016.128)
Supplement: Supplementary information, Figure S6 — The clustering analysis of accessibility of the NDRs in mammalian PGCs. [file cr2016128x9.pdf]

Figure S6

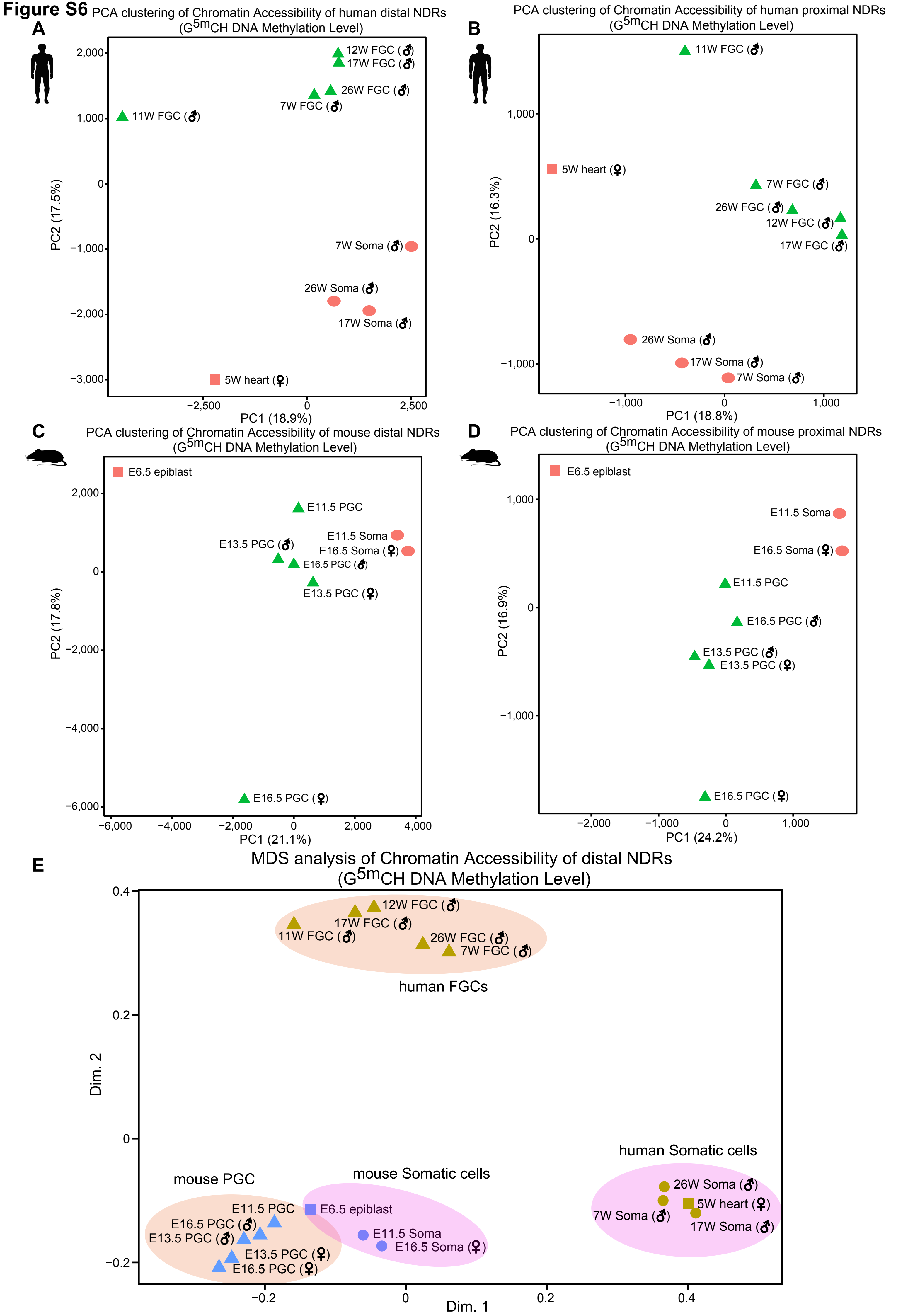
A
PCA clustering of Chromatin Accessibility of human distal NDRs
(G5mCH DNA Methylation Level)
12W FGC (♂)
17W FGC (♂)
26W FGC (♂)
7W FGC (♂)
11W FGC (♂)
7W Soma (♂)
26W Soma (♂)
17W Soma (♂)
5W heart (♀)
PC1 (18.9%)
PC2 (17.5%)
B
PCA clustering of Chromatin Accessibility of human proximal NDRs
(G5mCH DNA Methylation Level)
11W FGC (♂)
5W heart (♀)
7W FGC (♂)
26W FGC (♂)
12W FGC (♂)
17W FGC (♂)
26W Soma (♂)
17W Soma (♂)
7W Soma (♂)
PC1 (18.8%)
PC2 (16.3%)
C
PCA clustering of Chromatin Accessibility of mouse distal NDRs
(G5mCH DNA Methylation Level)
E6.5 epiblast
E11.5 PGC
E11.5 Soma
E13.5 PGC (♂)
E16.5 Soma (♀)
E16.5 PGC (♂)
E13.5 PGC (♀)
E16.5 PGC (♀)
PC1 (21.1%)
PC2 (17.8%)
D
PCA clustering of Chromatin Accessibility of mouse proximal NDRs
(G5mCH DNA Methylation Level)
E6.5 epiblast
E11.5 Soma
E16.5 Soma (♀)
E11.5 PGC
E16.5 PGC (♂)
E13.5 PGC (♂)
E13.5 PGC (♀)
E16.5 PGC (♀)
PC1 (24.2%)
PC2 (16.9%)
E
MDS analysis of Chromatin Accessibility of distal NDRs
(G5mCH DNA Methylation Level)
12W FGC (♂)
17W FGC (♂)
11W FGC (♂)
26W FGC (♂)
7W FGC (♂)
human FGCs
human Somatic cells
26W Soma (♂)
5W heart (♀)
7W Soma (♂)
17W Soma (♂)
mouse PGC
mouse Somatic cells
E11.5 PGC
E6.5 epiblast
E16.5 PGC (♂)
E13.5 PGC (♂)
E11.5 Soma
E16.5 Soma (♀)
E13.5 PGC (♀)
E16.5 PGC (♀)
Dim. 1
Dim. 2

Figure S6 The clustering analysis of accessibility of the NDRs in mammalian PGCs.

(A-D) PCA clustering of the distal (panel A and C) and proximal (panel B and D) NDRs based on the chromatin accessibilities in human (panel A and B) and mouse (panel C and D) samples. (E) The MDS analysis of chromatin accessibility of the distal NDRs of human-to-mouse homologous regions.
