# Supplementary material for: DNA methylation and chromatin accessibility profiling of mouse and human fetal germ cells
Source: Cell Res. 2016 Nov 8;27(2):165–83. doi: 10.1038/cr.2016.128 (PMC5339845; doi:10.1038/cr.2016.128)
Supplement: Supplementary information, Figure S7 — Chromatin accessibility at annotated elements and repetitive elements in mouse. [file cr2016128x10.pdf]

Figure S7

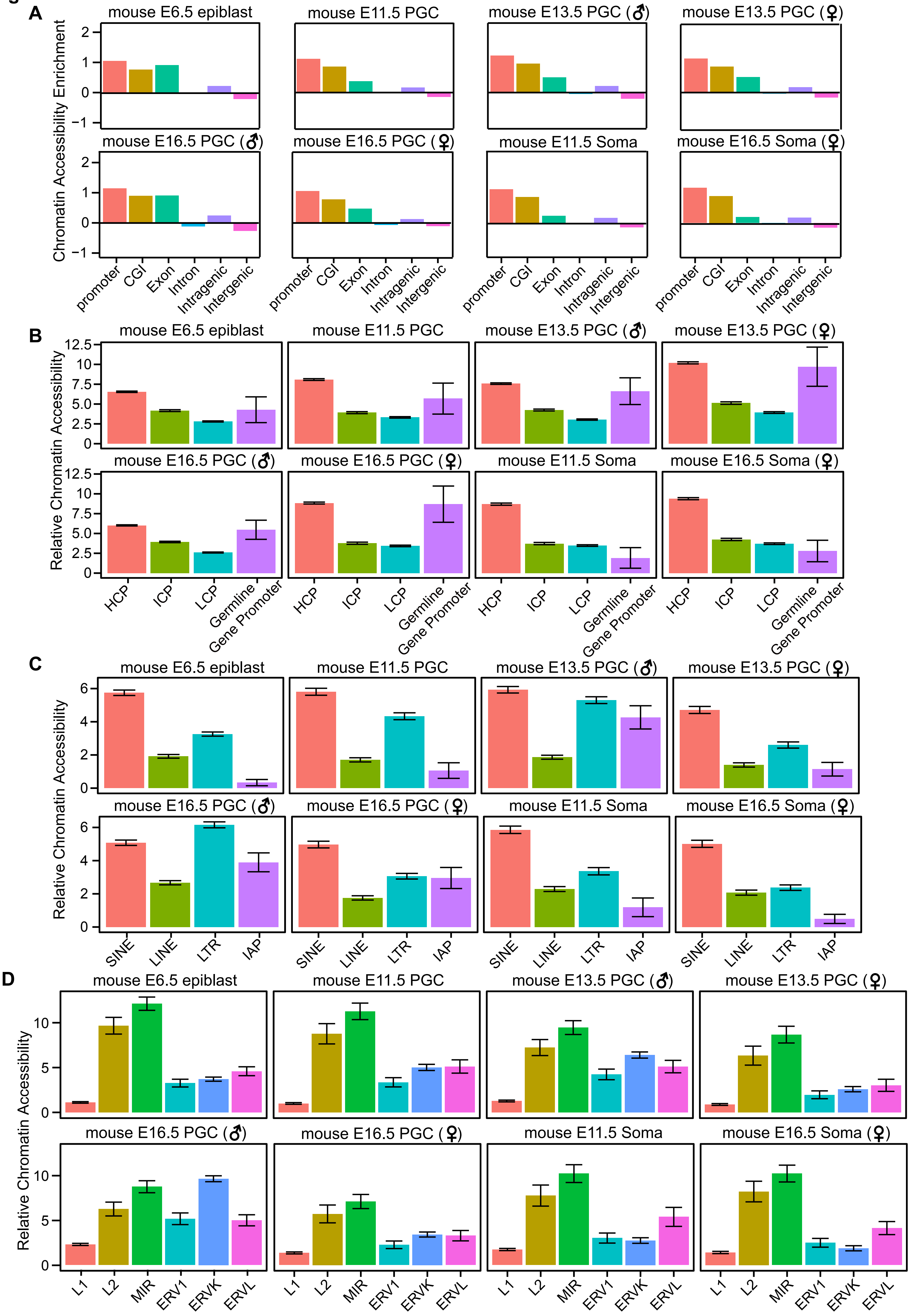
A
Chromatin Accessibility Enrichment
mouse E6.5 epiblast
mouse E11.5 PGC
mouse E13.5 PGC (♂)
mouse E13.5 PGC (♀)
mouse E16.5 PGC (♂)
mouse E16.5 PGC (♀)
mouse E11.5 Soma
mouse E16.5 Soma (♀)
promoter
CGI
Exon
Intron
Intragenic
Intergenic
B
Relative Chromatin Accessibility
HCP
ICP
LCP
Germline Gene Promoter
C
Relative Chromatin Accessibility
SINE
LINE
LTR
IAP
D
Relative Chromatin Accessibility
L1
L2
MIR
ERV1
ERVK
ERVL

Figure S7 Chromatin accessibility at annotated elements and repetitive elements in mouse.

(A) The relative enrichment analysis of the chromatin accessibility of the genomic regions in mouse. (B) The chromatin accessibility of the high-density CPG promoter (HCP), intermediate-density CpG promoter (ICP), low-density CpG promoter (LCP) and promoter of germline specific genes in mouse germline. (C-D) The chromatin accessibility at the repetitive elements (panel C) and their subfamilies (panel D) with different evolutional ages in mouse germline.
