# Supplementary material for: DNA methylation and chromatin accessibility profiling of mouse and human fetal germ cells
Source: Cell Res. 2016 Nov 8;27(2):165–83. doi: 10.1038/cr.2016.128 (PMC5339845; doi:10.1038/cr.2016.128)
Supplement: Supplementary information, Figure S8 — The relationships among chromatin accessibility, endogenous DNA methylation and gene expression of repetitive elements. [file cr2016128x11.pdf]

Figure S8

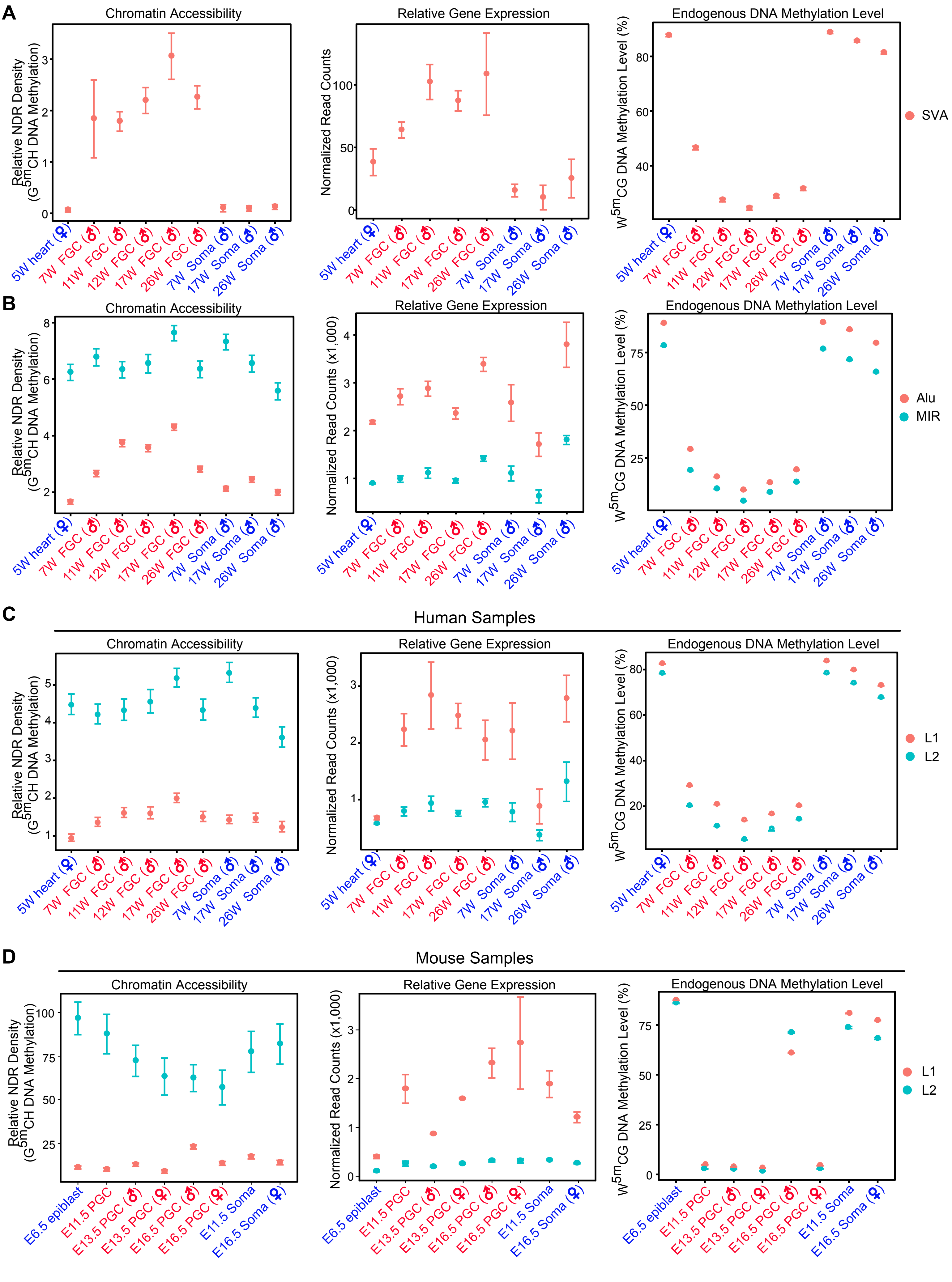
A
Chromatin Accessibility
Relative NDR Density
($G^{5m}CH$ DNA Methylation)
Relative Gene Expression
Normalized Read Counts
Endogenous DNA Methylation Level
$W^{5m}CG$ DNA Methylation Level (%)
SVA
5W heart (♀)
7W FGC (♂)
11W FGC (♂)
12W FGC (♂)
17W FGC (♂)
26W FGC (♂)
7W Soma (♂)
17W Soma (♂)
26W Soma (♂)
B
Chromatin Accessibility
Relative Gene Expression
Normalized Read Counts (x1,000)
Endogenous DNA Methylation Level
Alu
MIR
C
Human Samples
Chromatin Accessibility
Relative Gene Expression
Endogenous DNA Methylation Level
L1
L2
D
Mouse Samples
Chromatin Accessibility
Relative Gene Expression
Endogenous DNA Methylation Level
L1
L2
E6.5 epiblast
E11.5 PGC
E13.5 PGC (♂)
E13.5 PGC (♀)
E16.5 PGC (♂)
E16.5 PGC (♀)
E11.5 Soma
E16.5 Soma (♀)

Figure S8 The relationships among chromatin accessibility, endogenous DNA methylation and gene expression of repetitive elements.

(A) The relationship among chromatin accessibility, gene expression and endogenous DNA methylation of SVA element in human PGCs. (B) The relationship among chromatin accessibility, gene expression and endogenous DNA methylation of Alu (red) and MIR (cyan) repeat in human PGCs. (C-D) The relationship among chromatin accessibility, gene expression and endogenous DNA methylation of L1 (red) and L2 (cyan) repeat in human (panel C) and mouse (panel D) PGCs.
