# Supplementary material for: DNA methylation and chromatin accessibility profiling of mouse and human fetal germ cells
Source: Cell Res. 2016 Nov 8;27(2):165–83. doi: 10.1038/cr.2016.128 (PMC5339845; doi:10.1038/cr.2016.128)
Supplement: Supplementary information, Figure S9 — The relationships between the histone modifications and the chromatin accessibilities in mouse PGCs. [file cr2016128x12.pdf]

Figure S9

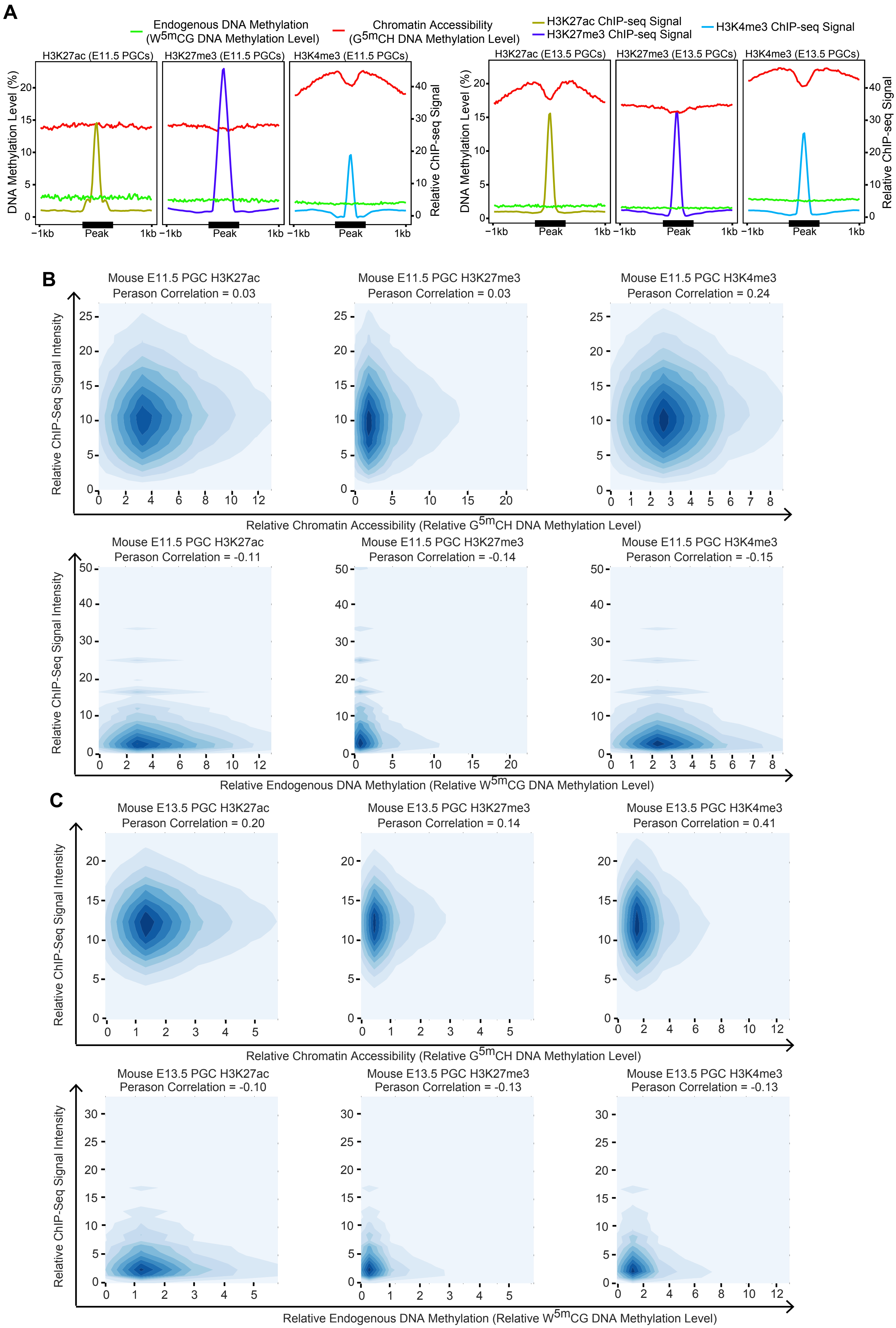

A
Endogenous DNA Methylation (W5mCG DNA Methylation Level)
Chromatin Accessibility (G5mCH DNA Methylation Level)
H3K27ac ChIP-seq Signal
H3K27me3 ChIP-seq Signal
H3K4me3 ChIP-seq Signal
H3K27ac (E11.5 PGCs)
H3K27me3 (E11.5 PGCs)
H3K4me3 (E11.5 PGCs)
H3K27ac (E13.5 PGCs)
H3K27me3 (E13.5 PGCs)
H3K4me3 (E13.5 PGCs)
DNA Methylation Level (%)
Relative ChIP-seq Signal
−1kb
Peak
1kb
B
Mouse E11.5 PGC H3K27ac
Perason Correlation = 0.03
Mouse E11.5 PGC H3K27me3
Perason Correlation = 0.03
Mouse E11.5 PGC H3K4me3
Perason Correlation = 0.24
Relative ChIP-Seq Signal Intensity
Relative Chromatin Accessibility (Relative G5mCH DNA Methylation Level)
Mouse E11.5 PGC H3K27ac
Perason Correlation = -0.11
Mouse E11.5 PGC H3K27me3
Perason Correlation = -0.14
Mouse E11.5 PGC H3K4me3
Perason Correlation = -0.15
Relative Endogenous DNA Methylation (Relative W5mCG DNA Methylation Level)
C
Mouse E13.5 PGC H3K27ac
Perason Correlation = 0.20
Mouse E13.5 PGC H3K27me3
Perason Correlation = 0.14
Mouse E13.5 PGC H3K4me3
Perason Correlation = 0.41
Relative Chromatin Accessibility (Relative G5mCH DNA Methylation Level)
Mouse E13.5 PGC H3K27ac
Perason Correlation = -0.10
Mouse E13.5 PGC H3K27me3
Perason Correlation = -0.13
Mouse E13.5 PGC H3K4me3
Perason Correlation = -0.13
Relative Endogenous DNA Methylation (Relative W5mCG DNA Methylation Level)

Figure S9 The relationships between the histone modifications and the chromatin accessibilities in mouse PGCs.

(A) The chromatin accessibility and endogenous DNA methylation level on the ChIP-seq peak and the flanking regions in mouse E11.5 and E13.5 PGCs. (B-C) The contour charts showing the person correlations between ChIP-seq signal of histone marks and the chromatin accessibility or endogenous DNA methylation in each consecutive 1kb bins in mouse E11.5 (panel B) and E13.5 (panel C) PGCs.
