# Supplementary material for: DNA methylation and chromatin accessibility profiling of mouse and human fetal germ cells
Source: Cell Res. 2016 Nov 8;27(2):165–83. doi: 10.1038/cr.2016.128 (PMC5339845; doi:10.1038/cr.2016.128)
Supplement: Supplementary information, Figure S10 — The relationships between the DNA hydroxymethylation and the chromatin accessibility in human fetal germ cells. [file cr2016128x13.pdf]

Figure S10

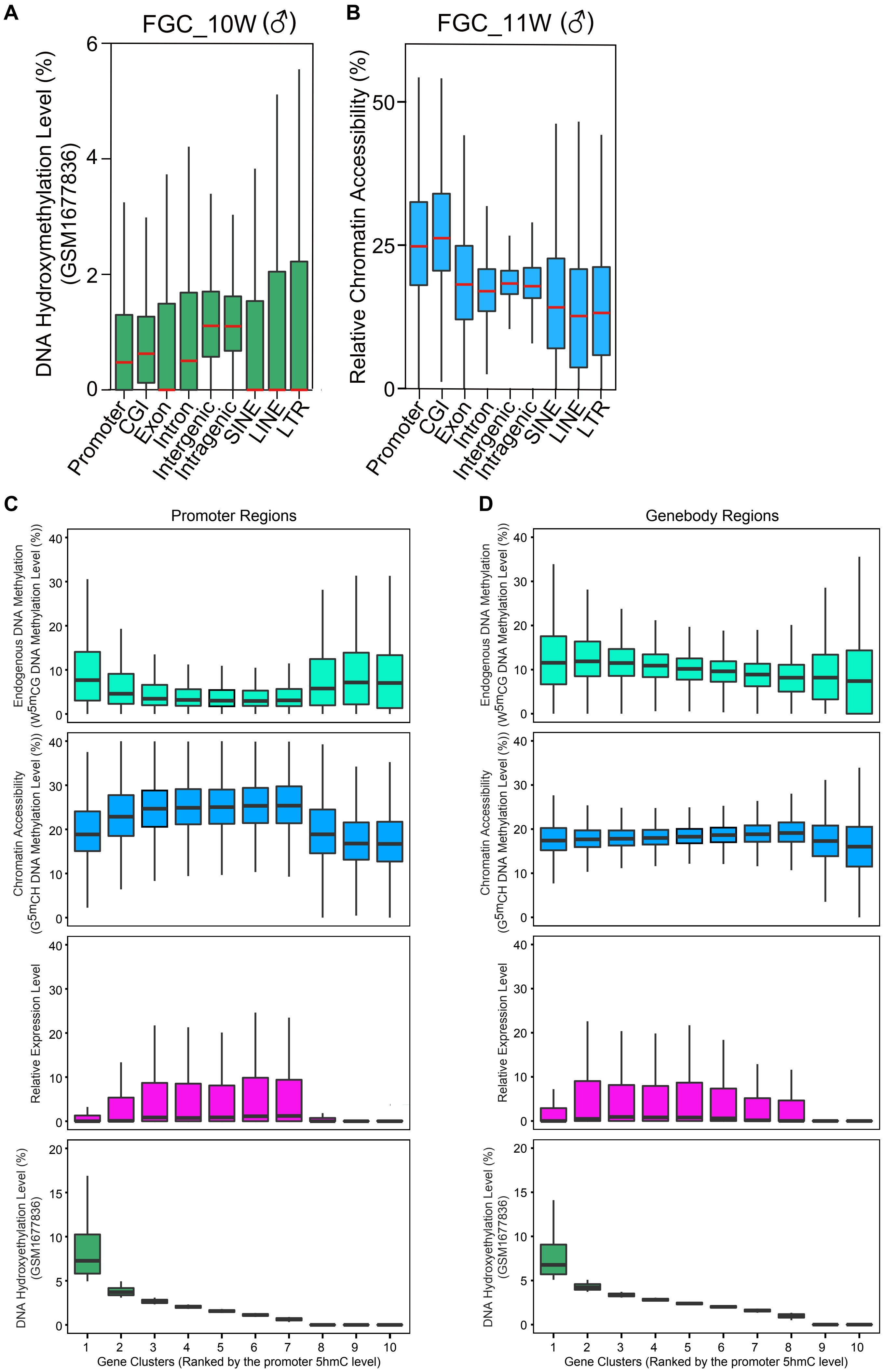
A
FGC_10W (♂)
DNA Hydroxymethylation Level (%) (GSM1677836)
0
2
4
6
Promoter
CGI
Exon
Intron
Intergenic
Intragenic
SINE
LINE
LTR
B
FGC_11W (♂)
Relative Chromatin Accessibility (%)
0
25
50
Promoter
CGI
Exon
Intron
Intergenic
Intragenic
SINE
LINE
LTR
C
Promoter Regions
Endogenous DNA Methylation (W5mCG DNA Methylation Level (%))
Chromatin Accessibility (G5mCH DNA Methylation Level (%))
Relative Expression Level
DNA Hydroxyethylation Level (%) (GSM1677836)
0
10
20
30
40
0
5
10
15
20
1
2
3
4
5
6
7
8
9
10
Gene Clusters (Ranked by the promoter 5hmC level)
D
Genebody Regions
Endogenous DNA Methylation (W5mCG DNA Methylation Level (%))
Chromatin Accessibility (G5mCH DNA Methylation Level (%))
Relative Expression Level
DNA Hydroxyethylation Level (%) (GSM1677836)
Gene Clusters (Ranked by the promoter 5hmC level)

Figure S10 The relationships between the DNA hydroxymethylation and the chromatin accessibility in human fetal germ cells.

(A-B) The DNA hydroxymethylation levels (panel A) and the chromatin accessibilities (panel B) on annotated regions. (C-D) The relationships among the DNA hydroxymethylation, chromatin accessibility, endogenous DNA methylation and relative expression on gene promoter (panel C) and genebody (panel D) regions in human fetal germ cells.
