# Supplementary material for: DNA methylation and chromatin accessibility profiling of mouse and human fetal germ cells
Source: Cell Res. 2016 Nov 8;27(2):165–83. doi: 10.1038/cr.2016.128 (PMC5339845; doi:10.1038/cr.2016.128)
Supplement: Supplementary information, Figure S11 — The nucleosome patterning on the intron-exon boundary. [file cr2016128x14.pdf]

Figure S11

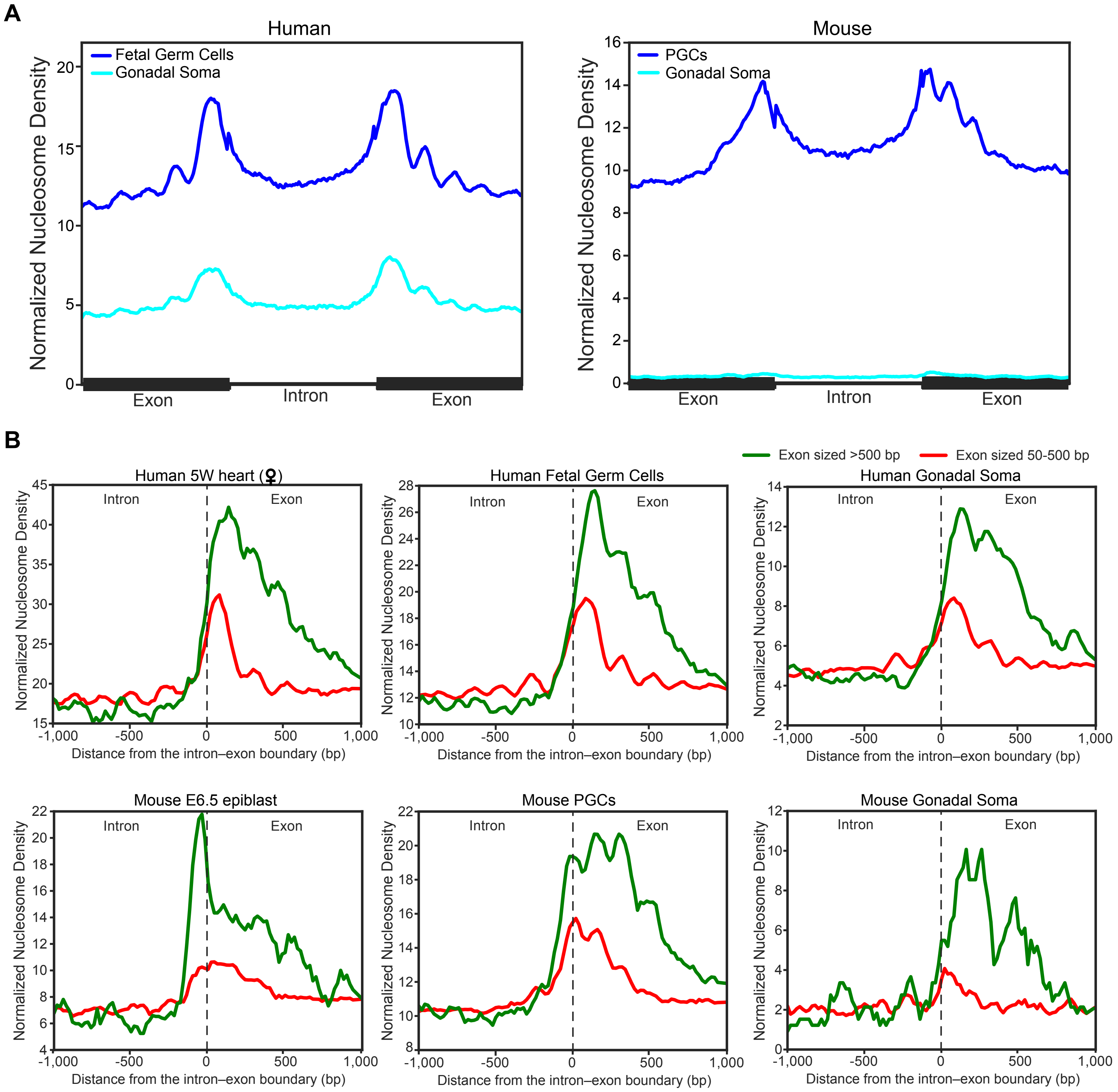

A
Human
Fetal Germ Cells
Gonadal Soma
Normalized Nucleosome Density
Exon
Intron
Exon
Mouse
PGCs
Gonadal Soma
Normalized Nucleosome Density
Exon
Intron
Exon
B
Exon sized >500 bp
Exon sized 50-500 bp
Human 5W heart (♀)
Human Fetal Germ Cells
Human Gonadal Soma
Mouse E6.5 epiblast
Mouse PGCs
Mouse Gonadal Soma
Intron
Exon
Normalized Nucleosome Density
Distance from the intron–exon boundary (bp)

Figure S11 The nucleosome patterning on the intron-exon boundary.
(A) The nucleosome strongly positioned on the intron-exon boundary in mammalian germlines. (B) The nucleosome patterns on the intron-exon boundary in human and mouse samples with different exon sizes.
